# Graveoumarins A–C: chiral resolution, absolute configuration, and anticoagulant/anti-inflammatory activities of 3′-methyl-3′-butenyl coumarins from *ruta graveolens* L

**DOI:** 10.1080/13880209.2025.2599599

**Published:** 2025-12-13

**Authors:** Zhihao Wu, Xiaolin Liao, Yuxin Wang, Jian Yin, Xu Feng, Lingfei Tong, Hao Huang, Yueping Jiang, Xiongjun Hou

**Affiliations:** ^a^ Department of Pharmacy, Xiangya Hospital, Central South University, Changsha, China; ^b^ Xiangya School of Pharmaceutical Sciences, Central South University, Changsha, China; ^c^ National Clinical Research Center for Geriatric Disorders, Xiangya Hospital, Central South University, Changsha, China; ^d^ Department of Clinical Pharmacy, Hunan University of Medicine General Hospital, Huaihua, Hunan, China; ^e^ Department of Pharmacy, Jiangxi Provincial People’s Hospital, The First Affiliated Hospital of Nanchang Medical College, Nanchang, China; ^f^ Jiangxi Province Key Laboratory of Pharmacology of Traditional Chinese Medicine, School of Pharmacy, Gannan Medical University, Ganzhou, China

**Keywords:** Ruta graveolens L, enantiomeric coumarins, chiral HPLC resolution, absolute configurations, activity evaluation

## Abstract

**Context:**

Several coumarins have been isolated from *Ruta graveolens* L., but the chirality of many remains uncharacterized or their absolute configurations unresolved.

**Objective:**

This study aimed to comprehensively separate and characterize the chirality of 3′-methyl-3′-butenylcoumarins from *R. graveolens* extracts, determine their absolute configurations, and evaluate their anticoagulant and anti-inflammatory activities.

**Materials and methods:**

Comprehensive chromatographic separation and chiral HPLC analysis were employed on the *R. graveolens* extract. The structures of isolated compounds were elucidated using extensive spectroscopic data analysis (HR-ESI-MS, NMR) and by comparing experimental circular dichroism (CD) spectra with calculated electronic circular dichroism (ECD) spectra. The anticoagulant and anti-inflammatory (specifically inhibition of nitric oxide (NO) production in lipopolysaccharide (LPS)-induced RAW 264.7 macrophages) activities of the isolated compounds were evaluated.

**Results:**

The study led to the isolation of two pairs of enantiomeric 3′-methyl-3′-butenylcoumarins, present in both equivalent and inequivalent ratios. This included two previously undescribed chiral 3′-methyl-3′-butenylcoumarins with specific absolute configurations [(+)-2′*R*-**2** and (–)-2′*S*-**3**] and one undescribed achiral 3′-methyl-3′-butenylcoumarin (**1**). Among the tested compounds, only the racemic mixture (±)-**3** exhibited moderate inhibition of NO production in the anti-inflammatory assay. No significant anticoagulant activity was reported for the compounds.

**Conclusions:**

This study successfully characterized the chirality and determined the absolute configurations of specific 3′-methyl-3′-butenylcoumarins from *R. graveolens*, including the discovery of three new compounds. While most isolated compounds lacked significant anticoagulant or anti-inflammatory activity in the tested models, racemic (±)-**3** showed moderate anti-inflammatory potential by inhibiting NO production. These findings provide new insights for the future development and utilization of coumarins from *R. graveolens*.

## Introduction

Medicinal plants represent fundamental sources of natural products, offering high chemical diversity and diverse pharmacological activities. They constitute an important reservoir of lead compounds for developing new drugs (Newman and Cragg 2020; Wan et al. 2023; Zhou et al. 2023; Zhang et al. 2024; Yang et al. 2024).

*Ruta graveolens* L. is a perennial plant within the Rutaceae family, originating in the Mediterranean region and now cultivated worldwide (Luo et al. 2024). It serves as an important source of traditional Chinese medicine in China. The Compendium of Materia Medica (Bencao Gangmu) documents its use, recording that the whole herb can be employed medicinally to remove blood stasis, cool blood, relieve swelling, and reduce fever (Flora of China Editorial Committee 1997). Essential oils, alkaloids, and phenylpropanoids—including simple phenylpropanoids, coumarins, and lignans—are reported to be the dominant chemical constituents in *R. graveolens* (Liu et al. 2023; Luo et al. 2024). These constituents, along with extracts derived from the plant, have been reported to possess diverse pharmacological activities. These include antibacterial, anti-inflammatory, anticancer, antiproliferative, antioxidant, fertility-regulating, and anthelmintic properties, as well as effects on the nervous system (Luo et al. 2024).

Coumarins represent an important class of bioactive compounds in *R. graveolens*, exhibiting antibacterial, anti-inflammatory, anticancer, central nervous system, and cardiovascular activities (Luo et al. 2024). However, few studies have investigated the enantiomers of *R. graveolens* coumarins or evaluated their biological activities. Our previous study reported two pairs of coumarins with unprecedented carbon skeletons isolated from *R. graveolens*. As part of a program to systematically study the chemical diversity of traditional Chinese medicines and their biological effects, we investigated *R. graveolens* extracts (Luo et al. 2024).

**Figure 1.**
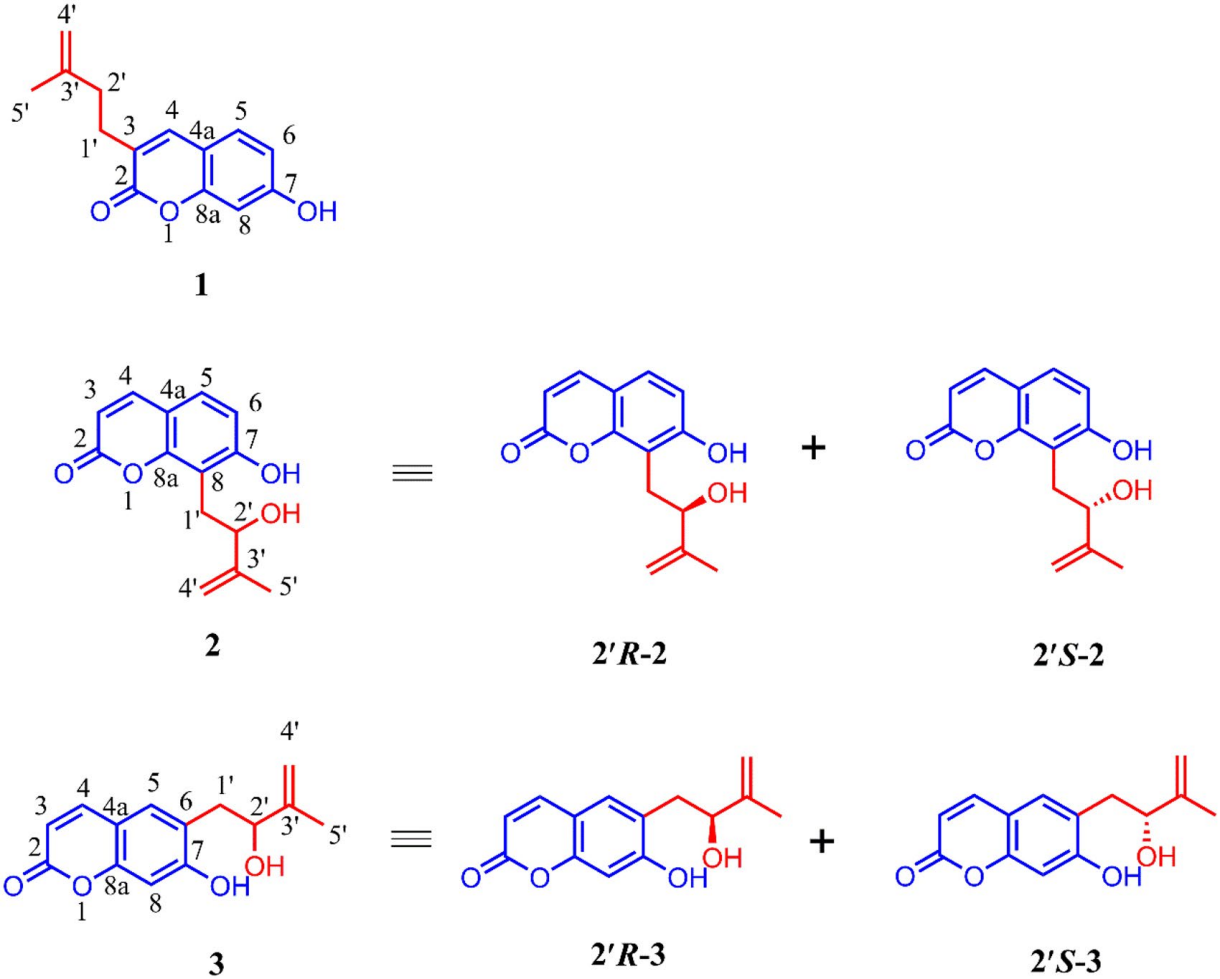
The structures of compounds **1**–**3**.

In the present study, we describe the isolation, chiral HPLC analysis, and structural identification of five coumarins, including two pairs of enantiomers. Among these, one coumarin features a previously undescribed structure, and the two enantiomeric pairs include compounds with previously unreported absolute configurations (Figure 1). We also evaluated the anticoagulant and anti-inflammatory activities of these compounds. Only racemic compound **3** [(±)-**3**] exhibited moderate anti-inflammatory activity.

## Materials and methods

### General experimental procedures

UV spectra were recorded on a Cary 300 UV-vis spectrometer. Optical rotations were measured using an AUTOPOL II automatic polarimeter equipped with a micro cell (10 mm path length; Rudolph Research Analytical, USA). CD spectra were acquired on a JASCO J-815 spectropolarimeter. IR spectra were recorded on a Nicolet IS50 FT-IR spectrometer (Thermo Fisher Scientific Co. Ltd., China). 1D and 2D NMR spectra were recorded in MeOD using a Bruker NMR spectrometer (AVANCE NEO 600 MHz; Bruker Ltd., Germany), with tetramethylsilane (TMS) as the internal reference. HR-ESI-MS data were acquired using an Agilent 1290 HPLC system (1290 Flexible Pump, G7114B DAD detector; Agilent Technologies Ltd., USA) coupled to an accurate-mass Q-TOF mass spectrometer (G6545B; Agilent Technologies Ltd., USA), employing a C18 column (3.0 × 150 mm, 1.8 μm; Agilent Technologies Ltd., USA). HPLC separation was performed on an Agilent system equipped with ChemStation software, a 1260 Quat Pump VL, a G7114A VWD absorbance detector, and a G1364F fraction collector (Agilent Technologies Ltd., USA), using YMC-Pack ODS-A (250 mm × 10 mm I.D.; YMC Co. Ltd., Japan) and YMC-Pack Phenyl (250 mm × 10 mm I.D.; YMC Co. Ltd., Japan) semi-preparative columns. Column chromatography (CC) was performed using silica gel (200–300 mesh; Qingdao Marine Chemical, Qingdao, China) and MCI gel CHP20P (Mitsubishi Chemical, Tokyo, Japan). Flash chromatography utilized Sephadex LH-20 (GE Healthcare, Sweden). Chiral HPLC analysis and preparation were conducted using a CHIRALPAK AD-H column (0.46 cm I.D. × 25 cm; Daicel Chiral Technologies Co. Ltd., China). TLC was performed on pre-coated silica gel GF254 plates. Spots were visualized under UV light (254 or 365 nm) or by spraying with 10% H_2_SO_4_ in 90% EtOH followed by heating. Unless otherwise noted, all chemicals were purchased from commercial sources and used without further purification.

Murine RAW264.7 macrophages (CVCL_E1BI, Cat. No.1001015) were purchased from Shanghai Institute of Biological Sciences (Shanghai, China). RPMI-1640 medium (Cat. No.D5796) and NG-monomethyl-L-arginine (L-NMMA) (Cat. No.D4902) were purchased from Sigma-Aldrich (St. Louis, USA). Trypsin cell digestion solution (with EDTA) (Cat. No. C0201) and streptomycin (Cat. No. SV30010) were purchased from Beyotime Biotech Inc. (Shanghai, China). Fetal bovine serum(FBS)(Cat. No. 10099141) were purchased from Gibco Life Sciences (New York, USA). Phosphate Buffered Saline (Cat. No. AWC0409) was purchased from Abiowell Biotechnology Co., Ltd. (Changsha, China).

### Plant material

The cultivated *R. graveolens* (10 kg) were collected from Guangning County (23°67’N, 112°20’E; Zhaoqing City, Guangdong Province, China) for experimental analysis. This study complies with local legislation. This plant is not protected, and the local government permits harvesting. Botanical authentication was performed by Prof. Shao Liu of Xiangya Hospital, Central South University, with voucher specimens (ID 2,020,001) archived in the Medicinal Chemistry Laboratory at Xiangya Hospital’s Pharmacy Building for long-term preservation and future. The Medicinal Chemistry Laboratory at the Xiangya Hospital Pharmacy Building is located on 87 Xiangya Road, Changsha 410008, China.

### Extraction and isolation

Extraction and initial Fractionation: The dried aerial parts of *R. graveolens* (10 kg) were powdered and extracted with water by the ultrasonic extraction method (8 L:1 *h* × 2 h) (Jiang et al. 2022, 2023). The aqueous extracts were combined and evaporated under reduced pressure to yield a dark-brown residue (1823.7 g). After aqueous extraction, the resulting herbal residue was then further extracted three times with 80% ethanol. The combined ethanol extracts were concentrated under reduced pressure to remove the ethanol. The concentrate was sequentially partitioned with petroleum ether and ethyl acetate. After concentration under reduced pressure, this process yielded three fractions: the petroleum ether-soluble fraction (YXC-A), the ethyl acetate-soluble fraction (YXC-B), and the remaining aqueous fraction (YXC-C).

Isolation and purification: The YXC-B fraction (approximately 80 g) was subjected to further isolation and purification. Initially, it was separated by silica gel column chromatography (CC), eluted with a gradient of dichloromethane (CH_2_Cl_2_) and methanol (MeOH) (0–100% MeOH), to yield nine subfractions (YXC-B1 to YXC-B9) based on thin-layer chromatography (TLC) analysis. Subfraction YXC-B4 (approximately 5 g) was subsequently separated by silica gel CC using a petroleum ether and ethyl acetate gradient (25–66.7% ethyl acetate), yielding eight fractions (YXC-B4-1 to YXC-B4-8). Fraction YXC-B4-6 (0.9 g) was further purified by Sephadex LH-20 column chromatography, eluted with CH_2_Cl_2_:MeOH (1:1), to afford six subfractions (YXC-B4-6-1 to YXC-B4-6-6).

Final purification and chiral resolution: Fraction YXC-B4-6-3 (approximately 20 mg) was purified by reversed-phase (RP) HPLC on a Phenomenex (Ph) column (2.0 mL/min) using 40% MeOH in H_2_O containing 0.1% formic acid (v/v) as the mobile phase, yielding compound **1** (3.0 mg, *t*_R_ = 28.6 min). Fraction YXC-B4-6-4 (approximately 30 mg) was purified by RP HPLC on a YMC-pack ODS-A column (2.0 mL/min) using 43% MeOH in H_2_O containing 0.1% formic acid (v/v), yielding compounds **2** (2.3 mg, *t*_R_ = 39.4 min) and **3** (6.9 mg, *t*_R_ = 53.5 min). To obtain the pure enantiomers, compound **2** was further separated by chiral HPLC on a CHIRALPAK AD-H column (0.8 mL/min) using 10% isopropanol in n-hexane as the mobile phase, yielding (–)-**2** (1.1 mg, *t*_R_ = 43.2 min) and (+)-**2** (0.8 mg, *t*_R_ = 47.5 min). Similarly, compound **3** was subjected to the same chiral HPLC conditions, yielding (–)-**3** (3.1 mg, *t*_R_ = 22.4 min) and (+)-**3** (3.0 mg, *t*_R_ = 27.0 min).

#### Graveoumarin A (1)

Yellow amorphous powder; UV (HPLC-UV in CH_3_CN/H_2_O) *λ*_max_ 238 nm, 323 nm; IR (KBr) *v*_max_ 3448, 1676, 1660, 1384, 1230 cm^–1^ (Newman and Cragg 2020); ^1^H and ^13^C NMR (MeOD) data, see Tables 1 and 2; (–)-HR-ESI-MS *m/z* 299.0868 [M–H]^–^ (calculated for C_14_H_13_O_3_, 299.0870)

**Table 1.** ^1^H NMR spectral data (*δ*, *J* in Hz) for compounds **1–3**.^*a*^

No.	1	2	3
3	–	6.17 d (*J* = 9.0 Hz)	6.16 d (*J* = 9.0 Hz)
4	7.64 s	7.83 d (*J* = 9.0 Hz)	7.82 d (*J* = 9.0 Hz)
4a	–	–	–
5	7.39 d (*J* = 8.4 Hz)	7.33 d (*J* = 8.4 Hz)	7.34 s
6	6.76 d (*J* = 8.4 Hz)	6.82 d (*J* = 8.4 Hz)	–
7	–	–	–
8	6.69 s	–	6.71 s
8a	–	–	–
1′a	2.63 t (*J* = 7.8 Hz)	3.16 dd (*J* = 13.2, 6.6 Hz)	2.94 dd (*J* = 13.8, 5.4 Hz)
1′b	–	3.06 dd (*J* = 13.2, 7.2 Hz)	2.79 dd (*J* = 13.8, 7.8 Hz)
2′	2.33 t (*J* = 7.8 Hz)	4.43 t (*J* = 6.6 Hz)	4.36 dd (*J* = 7.8, 5.4 Hz)
4′a	4.73 s	4.70 s	4.85 t (*J* = 0.6 Hz)
4′b	4.69 s	4.67 s	4.76 t (*J* = 0.6 Hz)
5′	1.79 s	1.85 s	1.79 s

^a^ 600 MHz for ^1^H NMR in CD_3_OD. Assignments were made by a combination of 1D and.

2D NMR experiments.

**Table 2.** ^13^C NMR spectral data (*δ*) for compounds **1–3**.^*a*^

No.	1	2	3
2	164.3	163.8	163.9
3	125.2	111.8	112.2
4	141.6	146.6	146.2
4a	113.7	113.3	112.9
5	129.9	128.4	131.7
6	114.3	113.9	125.4
7	162.1	161.4	161.4
8	103.0	114.3	103.0
8a	156.2	155.3	155.8
1′	29.9	30.1	37.4
2′	37.3	76.2	76.1
3′	146.0	148.4	148.6
4′	111.4	111.3	111.4
5′	22.5	17.7	18.0

^a^ 150 MHz for ^13^C NMR in CD_3_OD. Assignments were made by a combination of 1D and 2D NMR experiments.

#### (+)-/(–)-Graveoumarin B (2)

Yellow amorphous powder; [*α*]^25^_D_ +25.30 [*c* 0.03, CHCl_3_, (+)-**2**]; [*α*]^25^_D_ −26.03 [*c* 0.03, CHCl_3_, (–)-**2**]; UV (HPLC-UV in CH_3_CN/H_2_O) *λ*_max_ 222 nm, 258 nm, 327 nm; CD (MeOH) *λ*_max_ = 206.5 (mdeg −0.7365), 247.0 (mdeg −0.3223) nm, 269.0 (mdeg −0.2596) nm; [(+)-**2**]; CD (MeOH) *λ*_max_ = 200.0 (mdeg +2.6784), 311.5 (mdeg −0.2764) nm [(–)-**2**]; IR (KBr) *v*_max_ 3447, 2921, 2851, 1714, 1625, 1582, 1485, 1226, 1219, 1154, 1124 cm^–1^; ^1^H and ^13^C NMR (MeOD) data, see Tables 1 and 2; (+)-HR-ESI-MS *m/z* 247.0969 [M + H]^+^ (calculated for C_14_H_15_O_4_, 247.0965).

#### (+)-/(–)-Graveoumarin C (3)

Yellow amorphous powder; [*α*]^25^_D_ +19.38 [*c* 0.22, MeOH, (+)-**3**]; [*α*]^25^_D_ −21.87 [*c* 0.21, MeOH, (–)-**3**]; UV (HPLC-UV in CH_3_CN/H_2_O) *λ*_max_ 223 nm, 331 nm; CD (MeOH) *λ*_max_ = 206.5 (mdeg −2.4137), 225.5 (mdeg −1.0450) nm, 309.5 (mdeg −0.3779) nm; [(+)-**3**]; CD (MeOH) *λ*_max_ = 200.0 (mdeg +1.7762), 271.0 (mdeg −0.1787), 329.5 (mdeg +0.2258) nm [(–)-**3**]; IR (KBr) *v*_max_ 3338, 2918, 2850, 1704, 1623, 1573, 1392, 1274, 1136 cm^–1^; ^1^H and ^13^C NMR (MeOD) data, see Tables 1 and 2; (+)-HR-ESI-MS *m/z* 247.0970 [M + H]^+^ (calculated for C_14_H_15_O_4_, 247.0965).

### ECD calculation

Conformational analysis of compounds (2’*R*)-**2** and (2’*R*)-**3** was performed using the MMFF94 molecular mechanics force field within the MOE software package (Wang et al. 2019). Conformers with relative energies within 4 kcal/mol of the lowest-energy structure were optimized using density functional theory (DFT) at the B3PW91/TZVP level with empirical dispersion correction in methanol (MeOH). Energies, oscillator strengths, and rotational strengths for the first 30 electronic excitations were then calculated using time-dependent density functional theory (TDDFT) at the wB97XD/6-311 + G(d) level. ECD spectra for these conformers were simulated by applying a Gaussian function (σ = 0.35 eV). The final Boltzmann-weighted ECD spectrum for each compound was generated by summing the contributions of its respective conformers. All quantum chemical computations were performed using Gaussian 16 (Frisch et al. 2016).

### Biological activity assay

#### Anti-inflammatory assay

Murine RAW264.7 macrophages were obtained from the Cell Bank of the Chinese Academy of Sciences (Shanghai, China). Cells were cultured in RPMI-1640 medium supplemented with 10% fetal bovine serum (FBS), 100 U/mL penicillin, and 100 μg/mL streptomycin at 37 °C in a humidified atmosphere containing 5% CO_2_. To evaluate anti-inflammatory activity, cells were stimulated with lipopolysaccharide (LPS; 1 μg/mL) and simultaneously treated with the three test compounds for 48 h, using Dexamethasone as a positive control.

Nitric oxide (NO) production, reflecting inducible NO synthase (iNOS) activity, was measured by determining nitrite accumulation in the culture supernatant using the Griess reaction (Barger and Harmon 1997). Cell viability following treatment with various concentrations of the compounds was evaluated using the MTS assay. RAW264.7 macrophages were seeded into 96-well plates at a density of 8000 cells per well and incubated at 37 °C for 24 h. After the incubation period, the cells were treated with different concentrations of the compounds for another 24 h. According to the manufacturer’s instructions of the MTS assay kit (Beyotime Institute of Biotechnology, Shanghai, China), a working solution was prepared by adding MTS reagent to serum-free medium at a 10% (v/v) ratio. Then, 100 µL of this solution was added to each well, and the plates were incubated for 2 h in a cell culture incubator. The absorbance was measured at 570 nm using a SpectraMax microplate reader (Molecular Devices, Sunnyvale, CA, USA). Cell viability was calculated as follows: Cell viability (%) = [(As – Ab)/(Ac – Ab)] × 100, where As represents the absorbance of the test compound wells, Ab the absorbance of blank wells (medium without cells), and Ac the absorbance of control wells (cells treated with vehicle). All experiments were performed in triplicate, and the results were normalized to the LPS-treated control group, which was set to 100%.

#### Anticoagulant activity

The prothrombin time (PT) of compounds **1**–**3** was determined using a TECO MC-2000 coagulometer (Hamburg, Germany) with standard coagulation control plasma (TECO Medical Instruments, Niederbayern, Germany) and a commercial PT kit (Li et al., 2022). Briefly, each test compound (10 mM stock) was diluted to 1 mM in 0.22 M Tris-HCl buffer (pH 7.4), mixed with 45 μL of coagulation control plasma, and incubated at 37 °C for 2 min. Subsequently, 100 μL of pre-warmed PT reagent was added, and coagulation time was immediately recorded upon vigorous mixing.

## Results and discussion

### Structural elucidation

Compound **1** was obtained as a yellowish powder with C_14_H_14_O_3_ (8 degrees of unsaturation) using its HR-ESI-MS data (*m/z* 299.0868 [M–H]^–^, calculated for C_14_H_13_O_3_, 299.0870). The ^1^H NMR spectrum of **1** in CD_3_OD showed signals attributed to a meta-ortho-trisubstituted phenyl group [*δ*_H_ 7.39 (1H, d, *J* = 8.4 Hz, H-5), 6.76 (1H, dd, *J* = 8.4 Hz, H-6), and 6.69 (1H, s, H-8)], one olefin proton [*δ*_H_ 7.64 (1H, s, H-4),], two terminal olefin protons [4.73 (1H, s, H-4′a), 4.69 (1H, s, H-4′b)], two aliphatic methylenes [*δ*_H_ 2.63 (2H, t, *J* = 7.8 Hz, H-1′), and 2.33 (2H, d, *J* = 7.8 Hz, H-2′)], and one methyl group [1.79 (3H, s, H-5′)] (Tables 1 and 2); The ^13^C NMR and DEPT spectra of **1** showed carbon signals (Table 2) attributed to α,β-unsaturated lactone unit including one carbonyl [*δ*_C_ 164.3 (C-2)] and two olefin carbons [*δ*_C_ 141.6 (C-4) and 125.2 (C-3)], one meta-ortho-trisubstituted phenyl unit including two oxygen-bearing aromatic carbons [*δ*_C_ 162.1 (C-7) and 156.2(C-8a)], three proton-bearing proton aromatic carbons [*δ*_C_ 129.9 (C-5), 114.3 (C-6), and 103.0(C-8)], and one 3′-methyl-3′-butenyl group [*δ*_C_ 146.0 (C-3′), 111.4 (C-4′), 37.3 (C-2′), 29.9 (C-1′), and 22.5 (C-5′)]. These spectroscopic data suggested that **1** was a 7-hydroxylcoumarin with a 3′-methyl-3′-butenyl moiety substituted at C-3. The suggestion was supported by comparing the NMR data of compound **1** (which lacks a hydroxyl group at the C-2 position) with those of (2’*R*)-7-hydroxy-3-(2-hydroxy-3-methyl-3-butenyl) coumarin (Jian et al., 2024) and further confirmed by 2D NMR data analysis.

**Figure 2.**
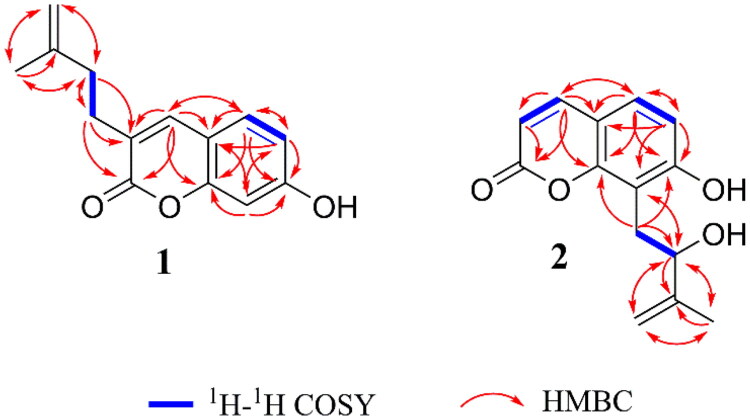
The key ^1^H-^1^H COSY and HMBC correlations of compounds **1**–**2**.

The ^1^H–^1^H COSY and HSQC spectra of **1** provided unambiguous assignments of proton and carbon signals in the NMR spectra. In the HMBC spectrum, from H-4 to C-2, C-4a, C-5, and C-8a; from H-5 to C-4, C-4a, C-6, C-7, and C-8a; from H-6 to C-4a, C-5, C-7, and C-8; and from H-8 to C-4a, C-8a, C-6, and C-7, together with their shifts and the molecular composition, demonstrated that the precent of coumarin moiety. In addition, HMBC correlations from H_2_-5′ and H_3_-4′ to C-2′; from H_2_-2′ and H_2_-1′ to C-3; from H_2_-1′ to C-2 and C-4 confirmed that the 3′-methyl-3′-butenyl unit was located at C-3 of the coumarin moiety (Figure 2). Therefore, the structure of compound **1** was determined as 7-hydroxy-3-(3′-methyl-3′-butenyl) coumarin based on the international union of pure and applied chemistry (IUPAC) nomenclature rules, named as being graveoumarin A.

Compound **2,** an enantiomeric mixture with [*α*]^25^_D_–5.8 (c 0.08, CHCl_3_), was isolated as a yellowish powder with C_14_H_14_O_4_ using its HR-ESI-MS data (*m/z* 247.0969 [M + H]^+^, calculated for C_14_H_15_O_4_, 247.0965). Comparison of the MS spectral data of **2** and **1** indicated that one more oxygen in **2** than that of in **1**. Comparison of the NMR spectral data **2** and **1** indicated that the aliphatic methylene [*δ*_H_ 2.33 (2H, d, *J* = 7.8 Hz, H-2′); *δ*_C_ 37.3 (C-2′)] in 3′-methyl-3′-butenyl unit of **1** was replaced by one oxygen-bearing methine [δ_H_ 4.43 (1H, t, *J* = 6.6 Hz, H-2′); δ_C_ 76.2 (C-2′)] in 3′-methyl-3′-butenyl unit of **2** (Tables 1 and 2). UV, IR, and NMR data analysis confirmed that **2** is a geometric isomer of (2′*R*)-7-hydroxy-3-(2-hydroxy-3-methyl-3-butenyl) coumarin (Jian et al., 2024). In particular, the HMBC correlations from H-2′ to C-8, H-1′ to C-7, C-8, and C-8a together with the chemical shifts of the methine unit (Tables 1 and 2), indicated that the 2′-hydroxy-3′-methyl-3′-butenyl was located at C-8 in **2** instead of C-3 in (2′*R*)-7-hydroxy-3-(2-hydroxy-3-methyl-3-butenyl) coumarin. The planar structure of **2** was the same of that of demethylauraptenol (Lee et al. 2014), and the result was confirmed by 2D NMR (Figure 2).

No significant cotton effect (CE) was observed in the experimental circular dichroism spectrum of **2**, indicating the isolation of a possible racemic mixture. Two chromatographic peaks with a near ratio of 2:3 were found by chiral HPLC analysis of **2** on a Chiralpak AD-H column. Compound (+)-**2** {[*α*]^25^_D_ = +25.30 (*c* 0.03, CHCl_3_)} and its enantiomer (−)-**2**{[*α*]^25^_D_=–26.03 (*c* 0.04, CHCl_3_)} were isolated by chiral HPLC using a chiral stationary phase. Their NMR spectra were identical, and their CD curves displayed mirror-image symmetry. As compound **2** contains only one chiral center, the absolute configuration of (+)-**2** was assigned as 2′*R* based on comparison of its specific optical rotation with that of the literature-reported (2′*R*)-7-hydroxy-3-(2-hydroxy-3-methyl-3-butenyl) coumarin (Jian et al. 2024) and auraptenol (Barik et al. 1983).

However, it must be noted that although the literature (Lee et al. 2014) established the planar structure and assigned the absolute configuration of demethylauraptenol as 2′S, this publication did not report the compound’s NMR data, specific optical rotation ([α]^25^_D_), or CD spectrum. Consequently, its chiroptical properties (specific optical rotation and CD) cannot be correlated with its assigned stereochemistry. Furthermore, the authors reporting the compounds demethylauraptenol (Lee et al. 2014) and (*R*)-(+)-7-hydroxy-8-(2-hydroxy-3-methyl-3-butenyl)-2*H*-1-benzopyran-2-one (Wu et al. 2003) misattributed their source reference. The purported original literature (Barik et al. 1983) cited for these compounds does not actually contain either compound or their corresponding NMR data. Therefore, Consequently, no literature reports exist providing the NMR data and definitive structural/chiroptical characterization (including absolute configuration assignment) for demethylauraptenol and (*R*)-(+)-7-hydroxy-8-(2-hydroxy-3-methyl-3-butenyl)-2H-chromen-2-one, despite both structures having been reported in the literature.

**Figure 3.**
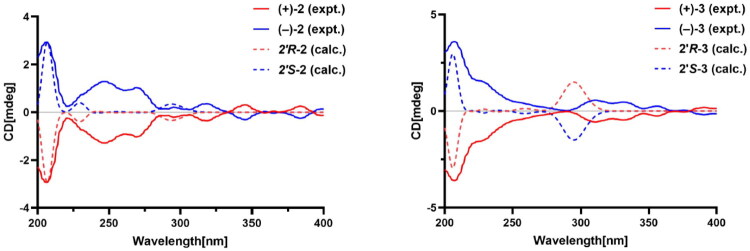
Experimental and calculated ECD spectra of compound **2**–**3.**

The absolute configuration of **2** was further established by comparison of the experimental and calculated electronic circular dichroism (ECD) spectra (Figure 3). Collectively, (+)-**2** and (–)-**2** were determined as being (+)-(2′*R*)-7-hydroxy-8-(2′-hydroxy-3′-methyl-3′-butenyl) coumarin and (–)-(2′*S*)-7-hydroxy-8-(2′-hydroxy-3′-methyl-3′-butenyl) coumarin, respectively. Thus, (+)-(2′*R*)-7-hydroxy-8-(2′-hydroxy-3′-methyl-3′-butenyl) coumarin and (–)-(2′*S*)-7-hydroxy-8-(2′-hydroxy-3′-methyl-3′-butenyl) coumarin named as (+)-graveoumarin B and (–)-graveoumarin B, respectively.

An enantiomeric mixture **3** was isolated as a yellow amorphous powder with C_14_H_15_O_4_ using its HR-ESI-MS data (*m/z* 247.0970 [M + H]^+^, calculated for C_14_H_15_O_4_, 247.0965), together with the NMR spectroscopic data (Table 1), which is homogeneous as indicated by reversed-phase high-performance liquid chromatography (RP-HPLC) analyses. Comparison of the UV, IR, MS and NMR spectral data of **3** and **2** indicated that **3** was the geometric isomer of **2**.

Comparison of the NMR data of **3** and **2** demonstrated that a 1,2,3,4-substituted aromatic ring of **2** was replaced by one 1,2,4,5-substituted aromatic ring in **3**. Therefore, two unimodal aromatic protons [*δ*_H_ 7.34 (1H, s, H-5) and 6.71(1H, s, H-8)] were observed in ^1^H NMR spectral of **3**. Additionally, an 2-hydroxy-3-methyl-3-butenyl unit signals [*δ*_H_ 2.94 (1H, dd, *J* = 13.8, 5.4 Hz, H-1′a), 2.79 (1H, dd, *J* = 13.8, 7.8 Hz, H-1′b), 4.36 (1H, dd, *J* = 7.8, 5.4 Hz, H-2′), 1.79 (3H, s, H-5′), 4.85 (1H, t, *J* = 0.6 Hz, H-4′a), and 4.76 (1H, d, *J* = 1,8 Hz, H-4′b); *δ*_C_ 148.6 (C-3′), 103.0 (C-8), 76.1 (C-2′), 37.4 (C-1′), and 18.0 (C-5′)] was also observed in NMR spectral of **3** (Tables 1 and 2). Comparison of the NMR data of **3** and [2-(*R*)-hydroxy-3-methyl-but-3-enyl]coumarin (Ahsan et al. 1994; Cheng et al. 2021) demonstrated that the 2′-hydroxy-3′-methyl-3′-butenyl unit was located at C-6 of coumarin. The planar structure of **3** was the same as that of [2-(*R*)–hydroxy-3-methyl–but-3-enyl] coumarin (Ahsan et al. 1994; Cheng et al. 2021).

HPLC analysis of **3** on an analytical chiral column displayed two peaks with a ratio of 1:1 integration, which proved that **3** was indeed a mixture of enantiomers in equivalent amounts. The absolute configuration of **3** was determined according to the optical rotation of [2-(*R*)–hydroxy-3-methyl–but-3-enyl] coumarin (Ahsan et al. 1994; Cheng et al. 2021), indicating the *R-* and *S*-configurations for (+)-**3** and (–)-**3**. Therefore, according to the IUPAC nomenclature rules, (+)-**3** and (–)-**3** were determined as being (+)-(2′*R*)-7-hydroxy-6-(2′-hydroxy-3′-methyl-3′-butenyl) coumarin and (–)-(2′*S*)-7-hydroxy-6-(2′-hydroxy-3′-methyl-3′-butenyl) coumarin, respectively. Thus, the two compounds were named as (+)-graveoumarin C and (–)-graveoumarin C, respectively.

### Biological evaluation

In the study, compounds **1**, (±)-**2**, and (±)-**3** were evaluated for anti-inflammatory and anticoagulant activities. No significant anticoagulant activity was observed for these compounds (prothrombin times: 12.8–13.4 s; *p* > 0.05 vs. control or positive control groups), compared to the control group and the positive control (Heparin: prothrombin time 21.6 ± 0.30 s).

In anti-inflammatory activity assays using RAW264.7 cells, **1** and (±)-**2** exhibited no significant inhibition of nitric oxide (NO) production (inhibition rates < 50%). However, (±)-**3** (5 µM) showed a NO inhibition rate of 51.67 ± 0.50%. Dexamethasone (5 µM), used as the positive control, exhibited a NO inhibition rate of 63.16 ± 2.27%. Further activity evaluation yielded the IC_50_ values of these compounds, as shown in Table 3.

**Table 3.** IC_50_ Values of the test compounds and positive control on NO inhibition in LPS-stimulated RAW 264.7 macrophages.

No.	IC_50_
Dexamethasone	2.92 μM
Compound **1**	9.36 μM
Compound (±)-**2**	6.88 μM
Compound (±)-**3**	4.68 μM

## Conclusions

In conclusion, two pairs of enantiomeric 3′-methyl-3′-butenylcoumarins in equivalent and inequivalent ratios, including two previously undescribed enantiomers, along with one undescribed achiral 3′-methyl-3′-butenylcoumarins, were isolated from the extract of *R. graveolens*. The enantiomers were separated by chiral HPLC, and their absolute configurations were determined by comparing ECD spectra and optical rotation data, corroborated by previous research and spectroscopic evidence. Biological evaluation revealed that none of the compounds exhibited anticoagulant activity. Only racemic **3** [(±)-**3**] demonstrated moderate anti-inflammatory activity. However, the biological activities of these compounds beyond those tested, as well as the anti-inflammatory mechanism of (±)-**3**, warrant further investigation. Additionally, the activities of the optically pure enantiomers obtained after chiral resolution require further evaluation. Due to the presence of equivalent and inequivalent coumarin enantiomers in *R. graveolens*, there is a significant risk of misassigning the absolute configuration of these compounds. Therefore, chiral configured coumarins must be analyzed by chiral HPLC.

## Supplementary Material

SI.docx

## Data Availability

All data included in this study are available upon request by contact with the corresponding author.
