# Supplementary material for: Graveoumarins A–C: chiral resolution, absolute configuration, and anticoagulant/anti-inflammatory activities of 3′-methyl-3′-butenyl coumarins from ruta graveolens L
Source: Pharm Biol. 2025 Dec 13;64(1):17–26. doi: 10.1080/13880209.2025.2599599 (PMC12704130; doi:10.1080/13880209.2025.2599599)
Supplement: SI.docx [file IPHB_A_2599599_SM1746.docx]

**Graveoumarins A–C: Chiral Resolution, Absolute Configuration, and Anticoagulant/Anti-inflammatory Activities of 3′-methyl-3′-butenylcoumarins from *Ruta graveolens* L.**

Zhihao Wu^a,b,c,1^, Xiaolin Liao^d,1^, Yuxin Wang ^a,b^, Jian Yin ^d^, Xu Feng ^a,b^, Lingfei Tong^e^, Hao Huang^f^, Yueping Jiang^a,c,d,^*, Xiongjun Hou^e,^*

*^a^* Department of Pharmacy, Xiangya Hospital, Central South University, Changsha 410008, China

*^b^* Xiangya School of Pharmaceutical Sciences, Central South University, Changsha 410013, China

*^c^*National Clinical Research Center for Geriatric Disorders, Xiangya Hospital, Central South University, Changsha 410008, China

*^d^*Department of Clinical Pharmacy, Hunan University of Medicine General Hospital, Huaihua, 418000, Hunan, China

*^e^*Department of Pharmacy, Jiangxi Provincial People's Hospital (The First Affiliated Hospital of Nanchang Medical College), Nanchang 330006, China

^f^Jiangxi Province Key Laboratory of Pharmacology of Traditional Chinese Medicine, School of Pharmacy, Gannan Medical University, Ganzhou 341000, China

^1^These authors contributed equally to this work.

*Correspondence: Yueping Jiang, [jiangyueping@csu.edu.cn](mailto:jiangyueping@csu.edu.cn) (Y. J.), +86 731 84327454

Xiongjun Hou, [66928053@qq.com](mailto:66928053@qq.com) (X.H.), +86 791 86891529

**Supporting Information**

**List of Contents**

| No. | Contents | Page |
| --- | --- | --- |
| 1 | **Figure S1**. The HPLC-UV spectrum of compound **1** in CH_3_CN/H_2_O. | S3 |
| 2 | **Figure S2**. The IR spectrum of compound **1**. | S3 |
| 3 | **Figure S3**. The (–)-HRESIMS report of compound **1**, page 1. | S4 |
| 4 | **Figure S4**. The (–)-HRESIMS report of compound **1**, page 2. | S5 |
| 5 | **Figure S5**. The ^1^H NMR spectrum of compound **1** in MeOH-*d*_4_ (600 MHz). | S6 |
| 6 | **Figure S6**. The ^13^C NMR spectrum of compound **1** in MeOH-*d*_4_ (150 MHz). | S6 |
| 7 | **Figure S7**. The DEPT spectrum of compound **1** in MeOH-*d*_4_ (150 MHz). | S7 |
| 8 | **Figure S8**. The ^1^H-^1^H COSY spectrum of compound **1** in MeOH-*d*_4_ (600 MHz). | S7 |
| 9 | **Figure S9**. The HSQC spectrum of compound **1** in MeOH-*d*_4_ (600 MHz for ^1^H). | S8 |
| 10 | **Figure S10**. The HMBC spectrum of compound **1** in MeOH-*d*_4_ (600 MHz for ^1^H). | S8 |
| 11 | **Figure S11**. The UV spectrum of compound **2** in MeOH. | S9 |
| 12 | **Figure S12**.The ECD spectrum of compound (+)-**2** in MeOH. | S9 |
| 13 | **Figure S13**. The ECD spectrum of compound (–)-**2** in MeOH. | S10 |
| 14 | **Figure S14**. The IR spectrum of compound **2**. | S11 |
| 15 | **Figure S15**. The (+)-HRESIMS report of compound **2**, page 1. | S12 |
| 16 | **Figure S16**. The (+)-HRESIMS report of compound **2**, page 2. | S12 |
| 17 | **Figure S17**. The ^1^H NMR spectrum of compound **2** in MeOH-*d*_4_ (600 MHz). | S13 |
| 18 | **Figure S18**. The ^13^C NMR spectrum of compound **2** in MeOH-*d*_4_ (150 MHz). | S14 |
| 19 | **Figure S19**. The DEPT spectrum of compound **2** in MeOH-*d*_4_ (150 MHz). | S14 |
| 20 | **Figure S20**. The ^1^H-^1^H COSY spectrum of compound **2** in MeOH-*d*_4_ (600 MHz). | S15 |
| 21 | **Figure S21**. The HSQC spectrum of compound **2** in MeOH-*d*_4_ (600 MHz for ^1^H). | S15 |
| 22 | **Figure S22**. The HMBC spectrum of compound **2** in MeOH-*d*_4_ (600 MHz for ^1^H). | S16 |
| 23 | **Figure S23**. The HPLC-UV spectrum of compound **3**. | S16 |
| 24 | **Figure S24**.The ECD spectrum of compound (+)-**3** in MeOH. | S17 |
| 25 | **Figure S25**. The ECD spectrum of compound (–)-**3** in MeOH. | S17 |
| 26 | **Figure S26**. The IR spectrum of compound **3**. | S18 |
| 27 | **Figure S27**. The (+)-HRESIMS report of compound **3**, page 1. | S19 |
| 28 | **Figure S28**. The (+)-HRESIMS report of compound **3**, page 2. | S20 |
| 29 | **Figure S29**. The ^1^H NMR spectrum of compound **3** in MeOH-*d*_4_ (600 MHz). | S21 |
| 30 | **Figure S30**. The ^13^C NMR spectrum of compound **3** in MeOH-*d*_4_ (150 MHz). | S21 |
| 31 | **Figure S31**. The DEPT spectrum of compound **3** in MeOH-*d*_4_ (150 MHz). | S22 |
| 32 | **Figure S32**. The ^1^H-^1^H COSY spectrum of compound **3** in MeOH-*d*_4_ (600 MHz). | S23 |
| 33 | **Figure S33**. The HSQC spectrum of compound **3** in MeOH-*d*_4_ (600 MHz for ^1^H). | S23 |
| 34 | **Figure S34**. The HMBC spectrum of compound **3** in MeOH-*d*_4_ (600 MHz for ^1^H). | S24 |

**Figure S1**. The HPLC-UV spectrum of compound **1** in CH_3_CN/H_2_O.

**

**Figure S2**. The IR spectrum of compound **1**.


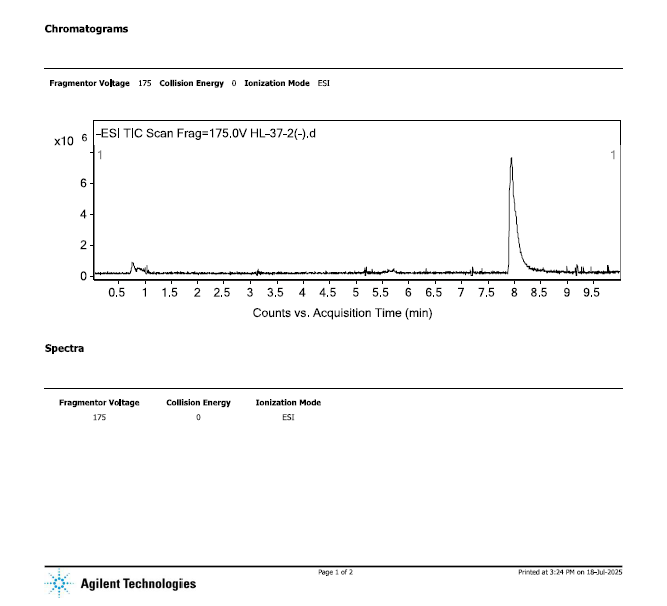


**Figure S3**. The (–)-HRESIMS report of compound **1**, page 1.


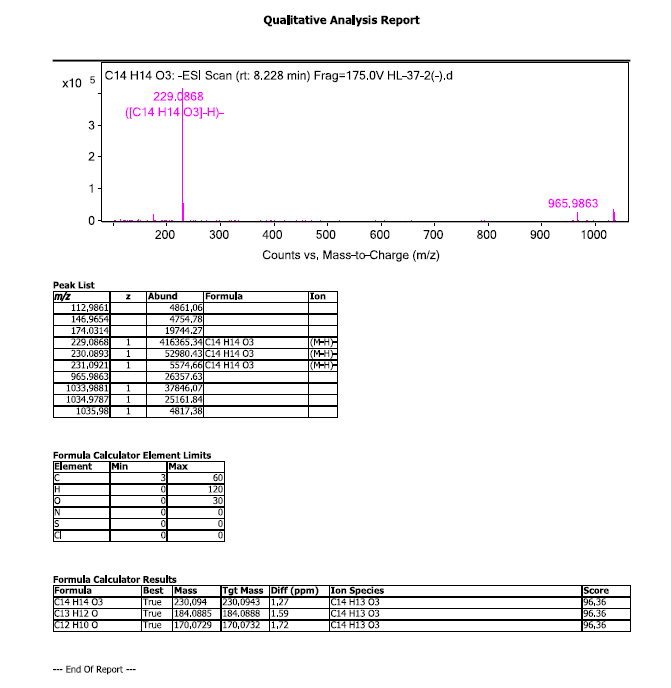


**Figure S4**. The (–)-HRESIMS report of compound **1**, page 2.


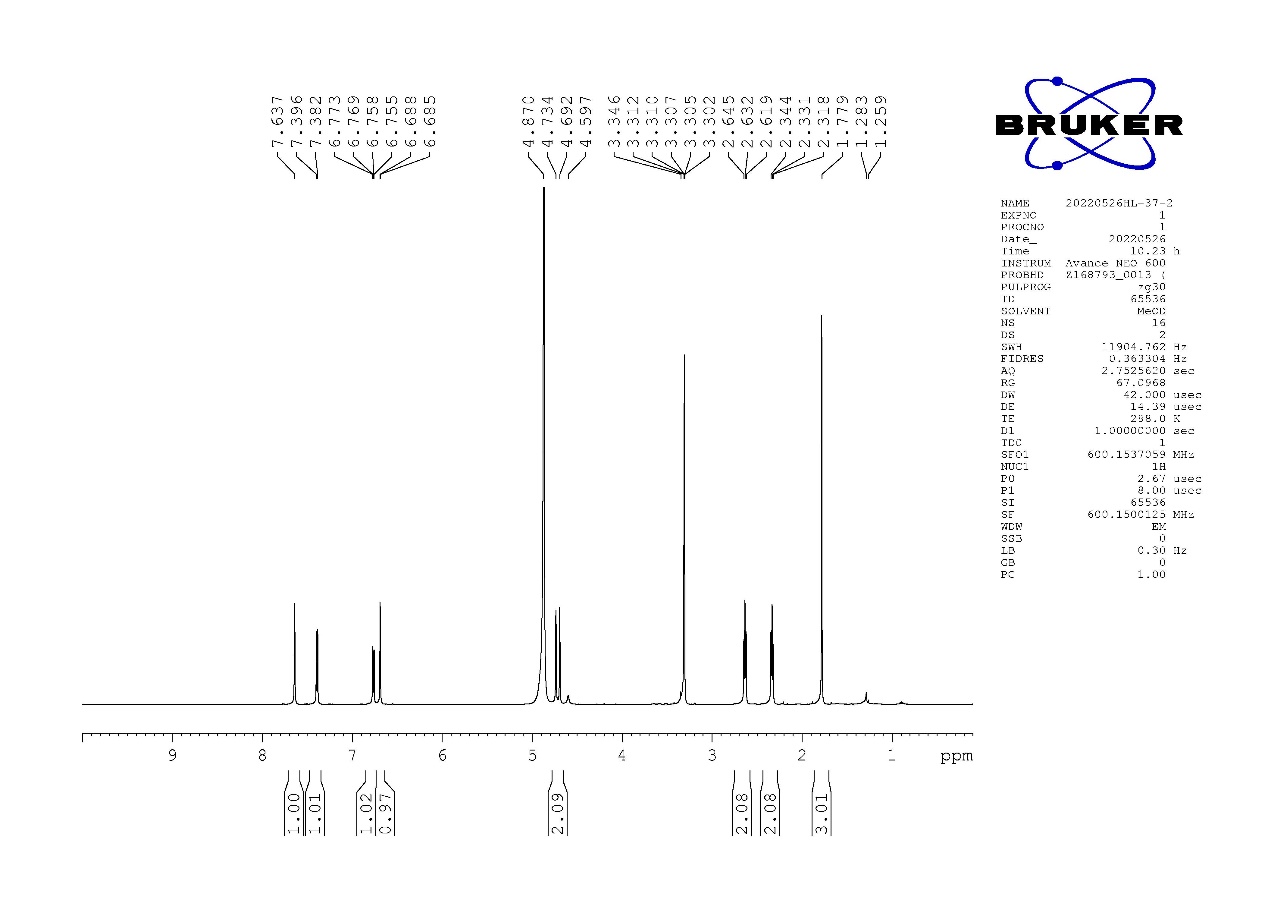


**Figure S5**. The ^1^H NMR spectrum of compound **1** in MeOH-*d*_4_ (600 MHz).


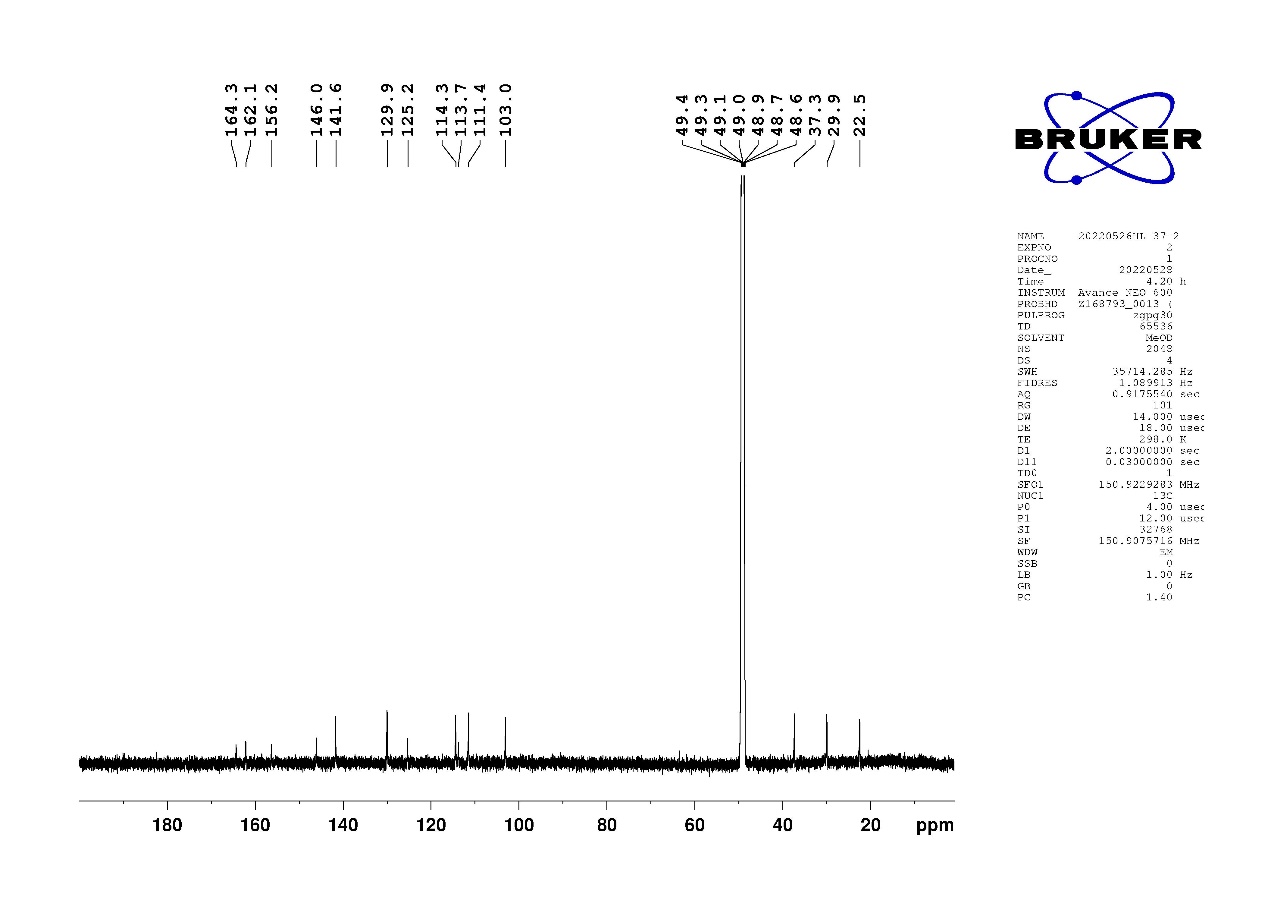


**Figure S6**. The ^13^C NMR spectrum of compound **1** in MeOH-*d*_4_ (150 MHz).


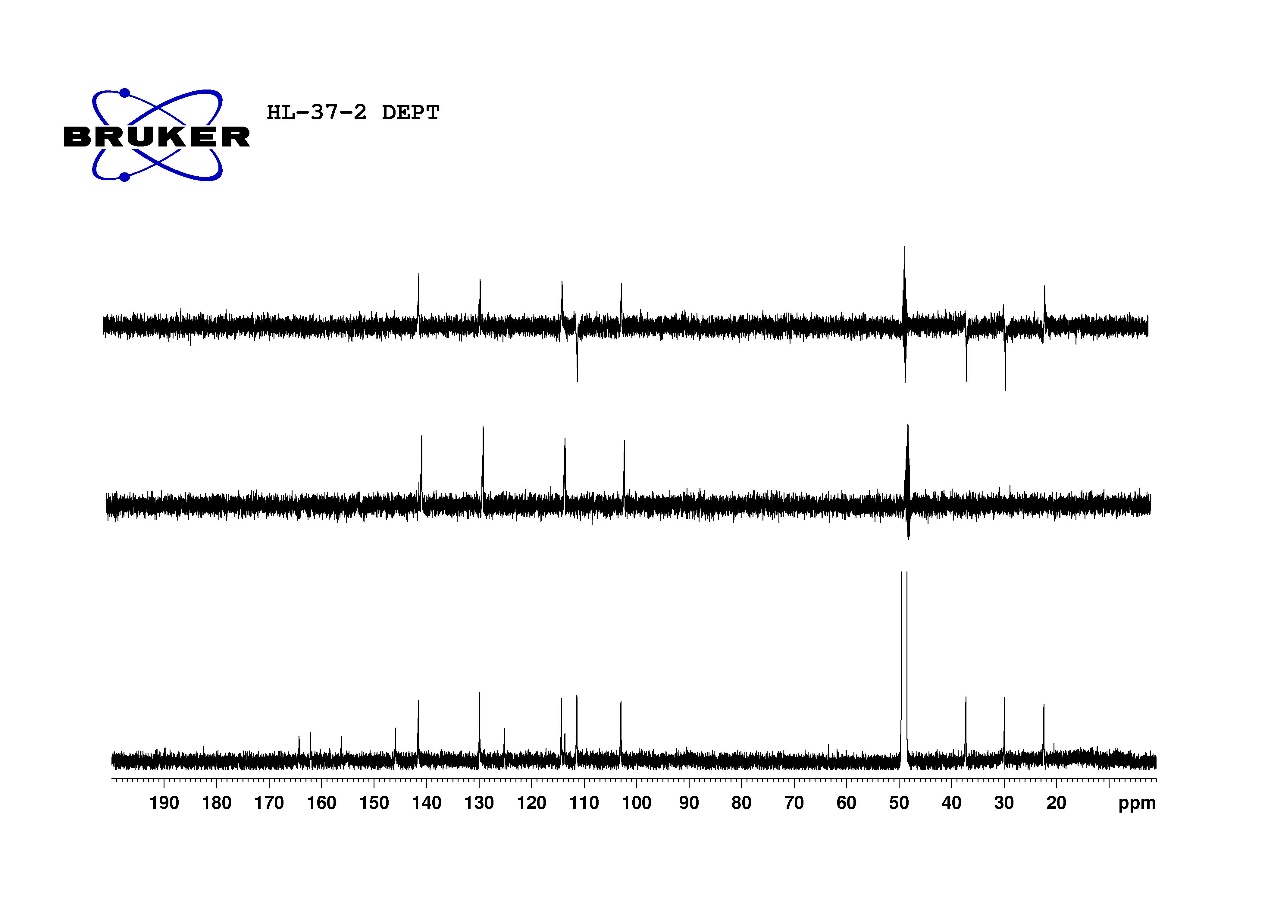


**Figure S7**. The DEPT spectrum of compound **1** in MeOH-*d*_4_ (150 MHz).


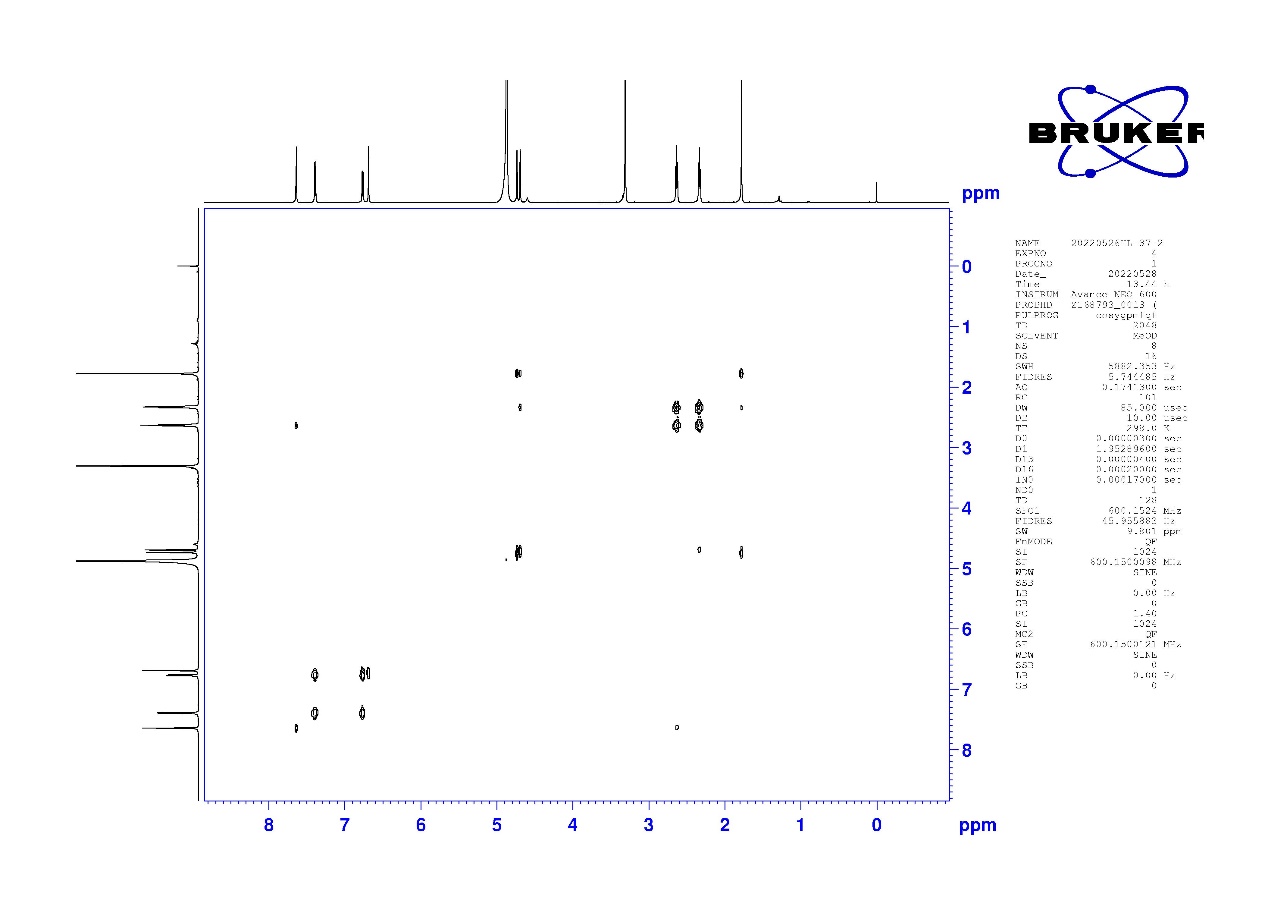


**Figure S8**. The ^1^H-^1^H COSY spectrum of compound **1** in MeOH-*d*_4_ (600 MHz).

**
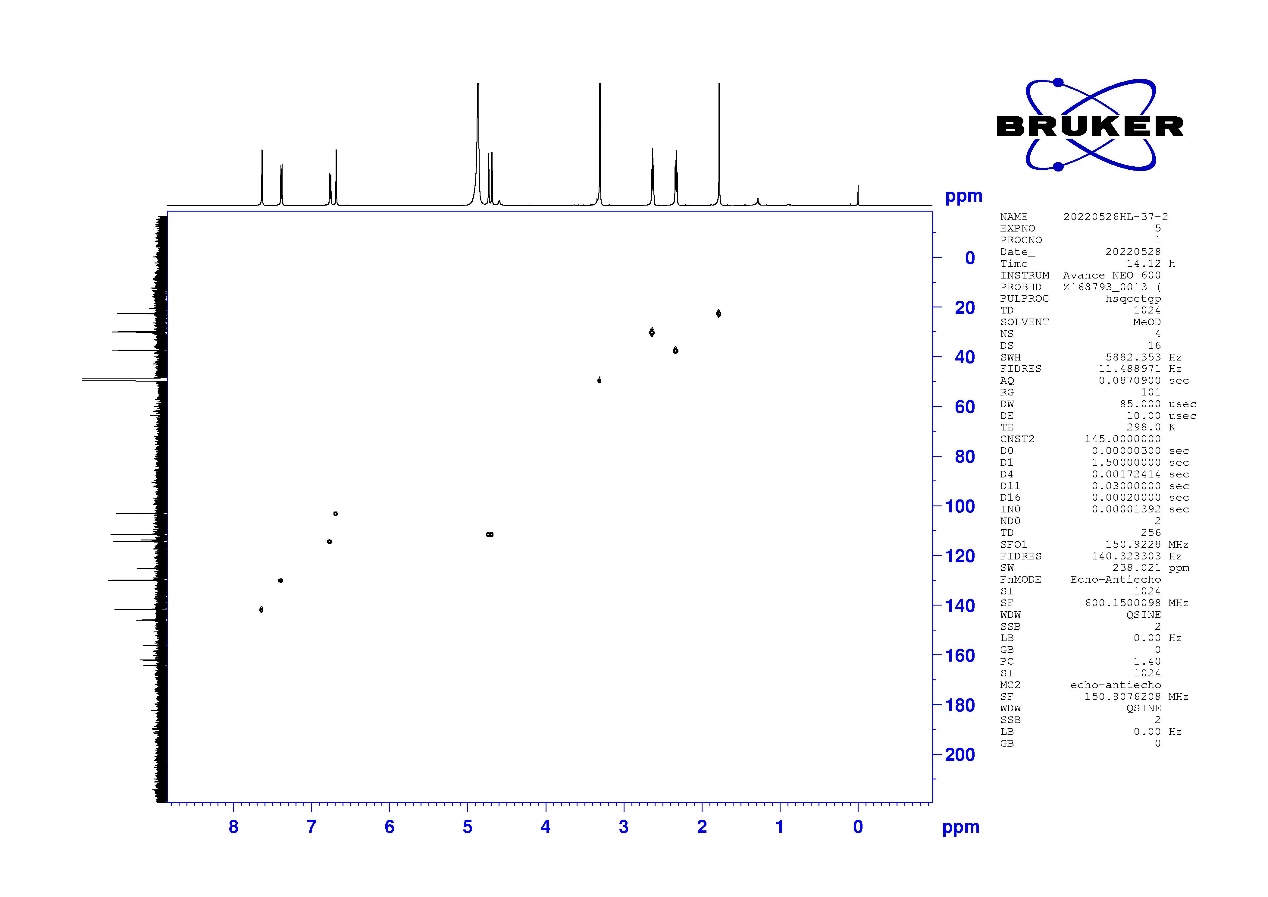
**

**Figure S9**. The HSQC spectrum of compound **1** in MeOH-*d*_4_ (600 MHz for ^1^H).


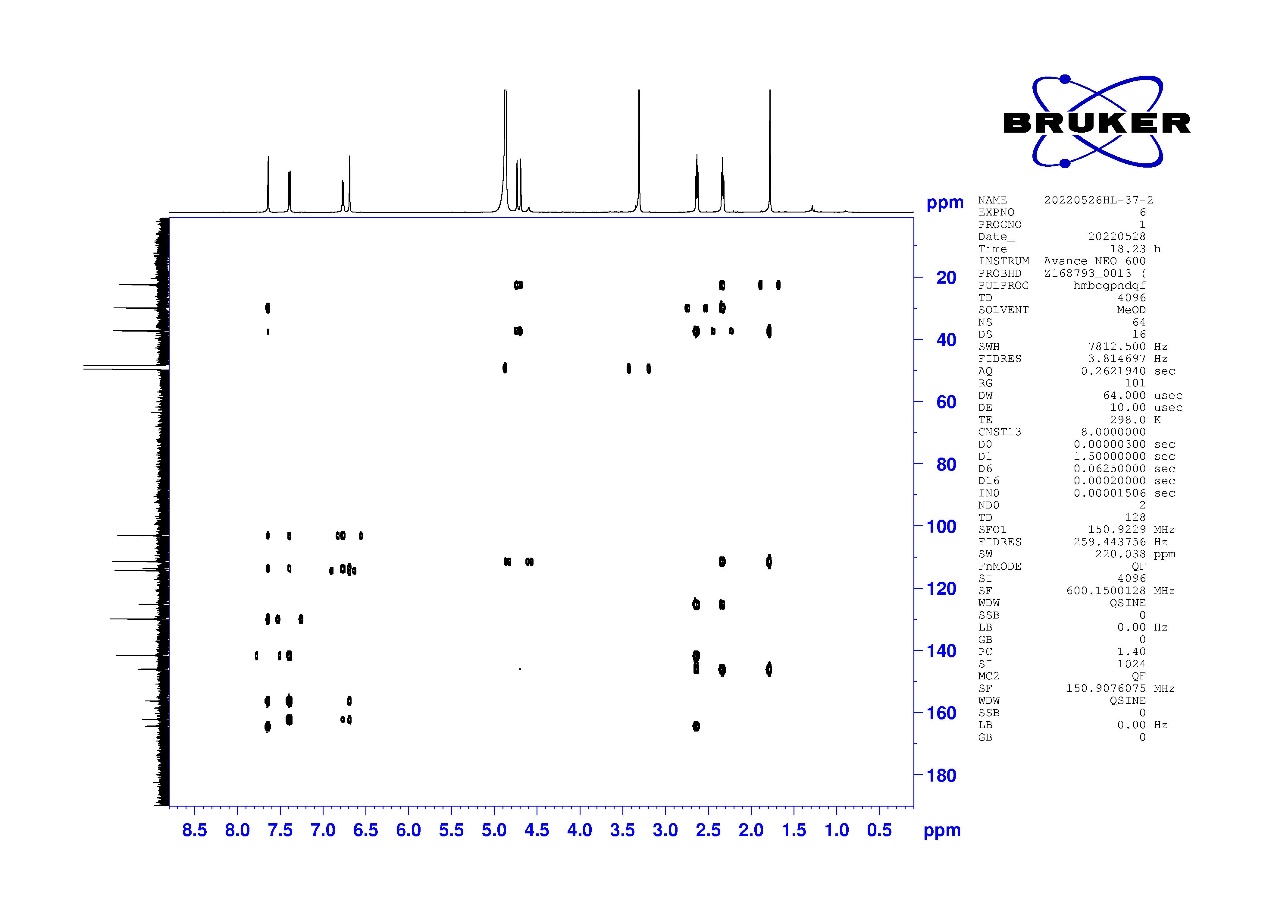


**Figure S10**. The HMBC spectrum of compound **1** in MeOH-*d*_4_ (600 MHz for ^1^H).

**Figure S11**. The chiral HPLC analysis of **2**.

**Figure S12**. The HPLC-UV spectrum of compound **2** in CH_3_CN/H_2_O.

**
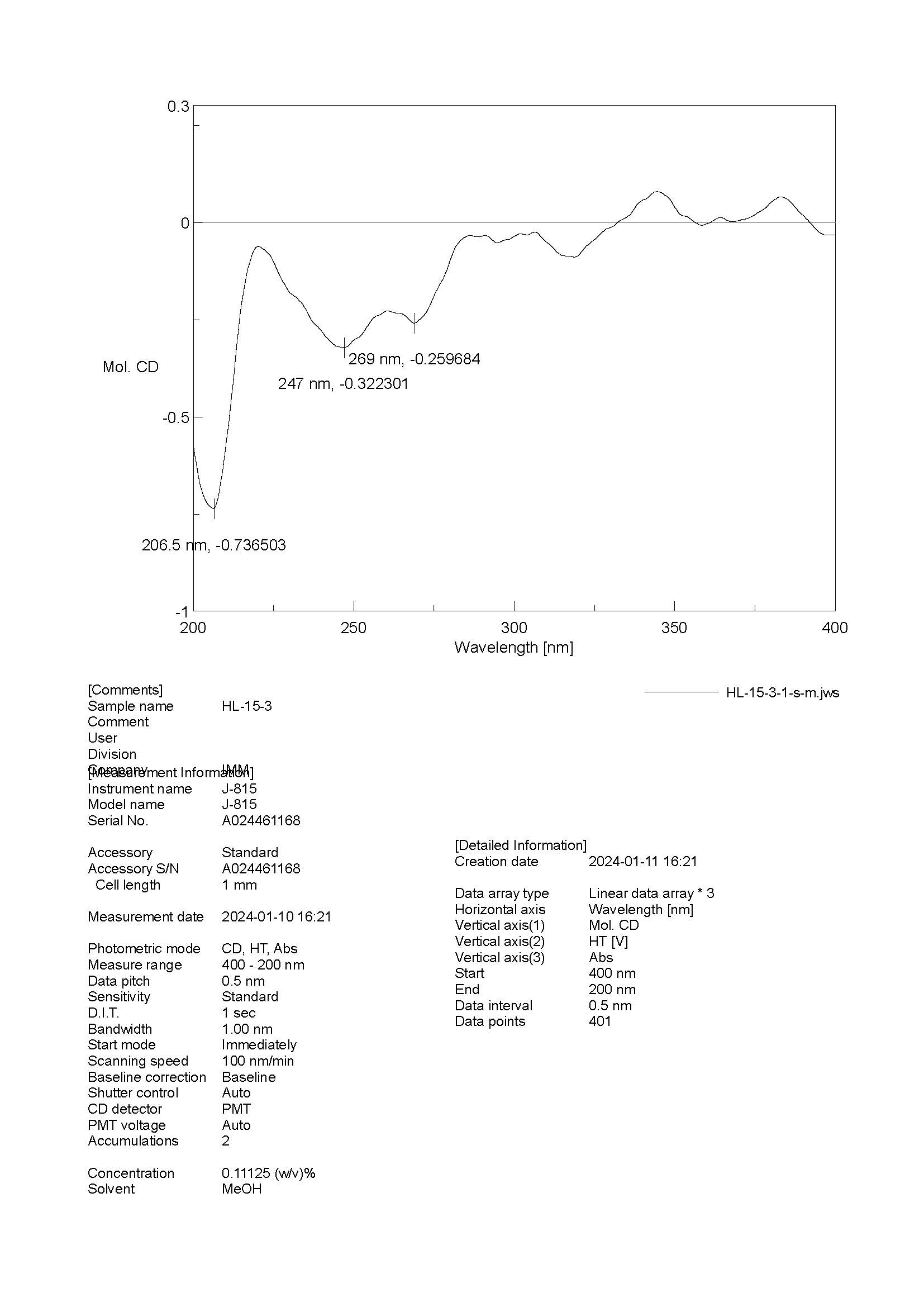
**

**Figure S13**. The CD spectrum of compound (+)-**2** in CH_3_OH.

**
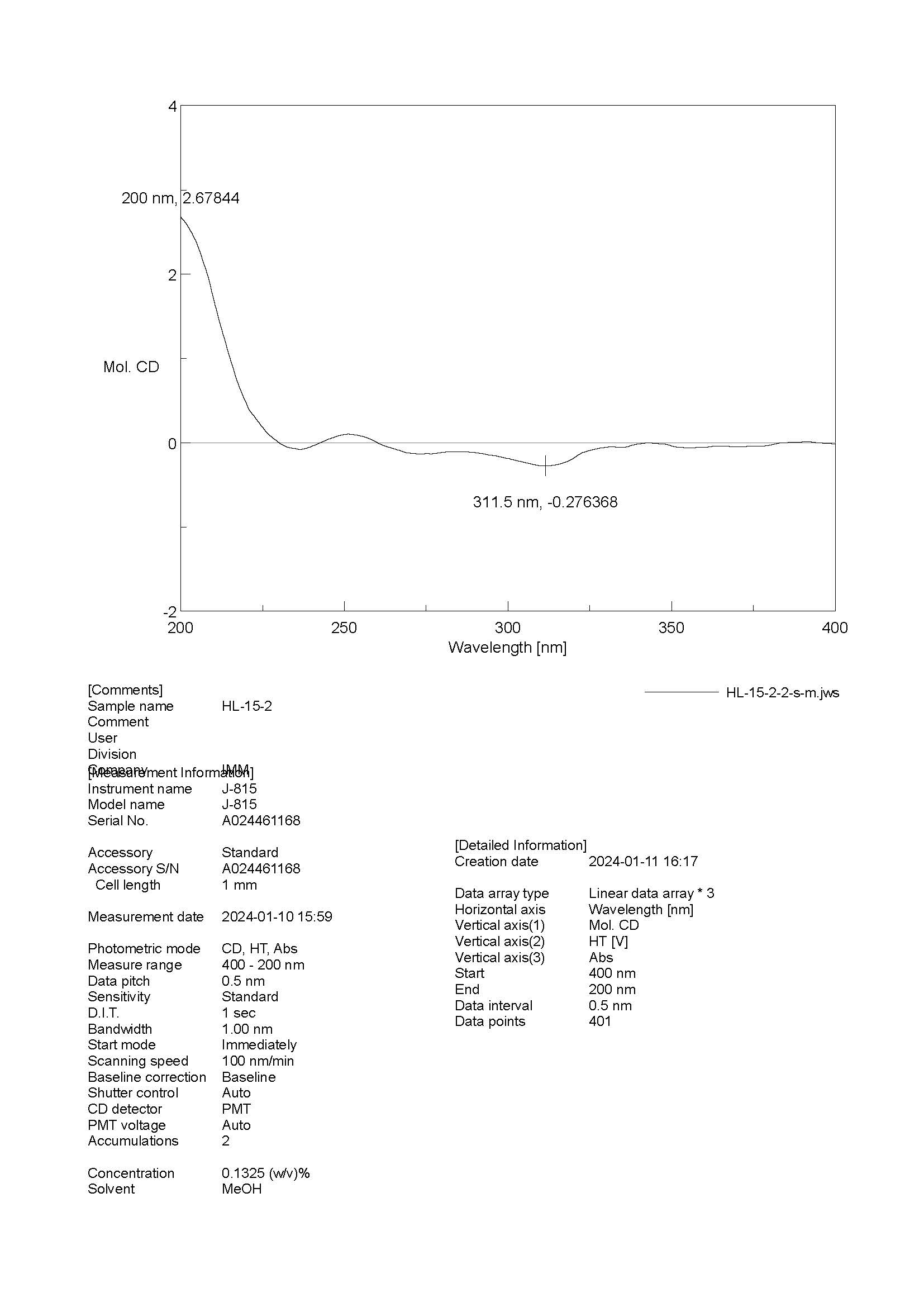
**

**Figure S14**. The CD spectrum of compound (–)-**2** in CH_3_OH.


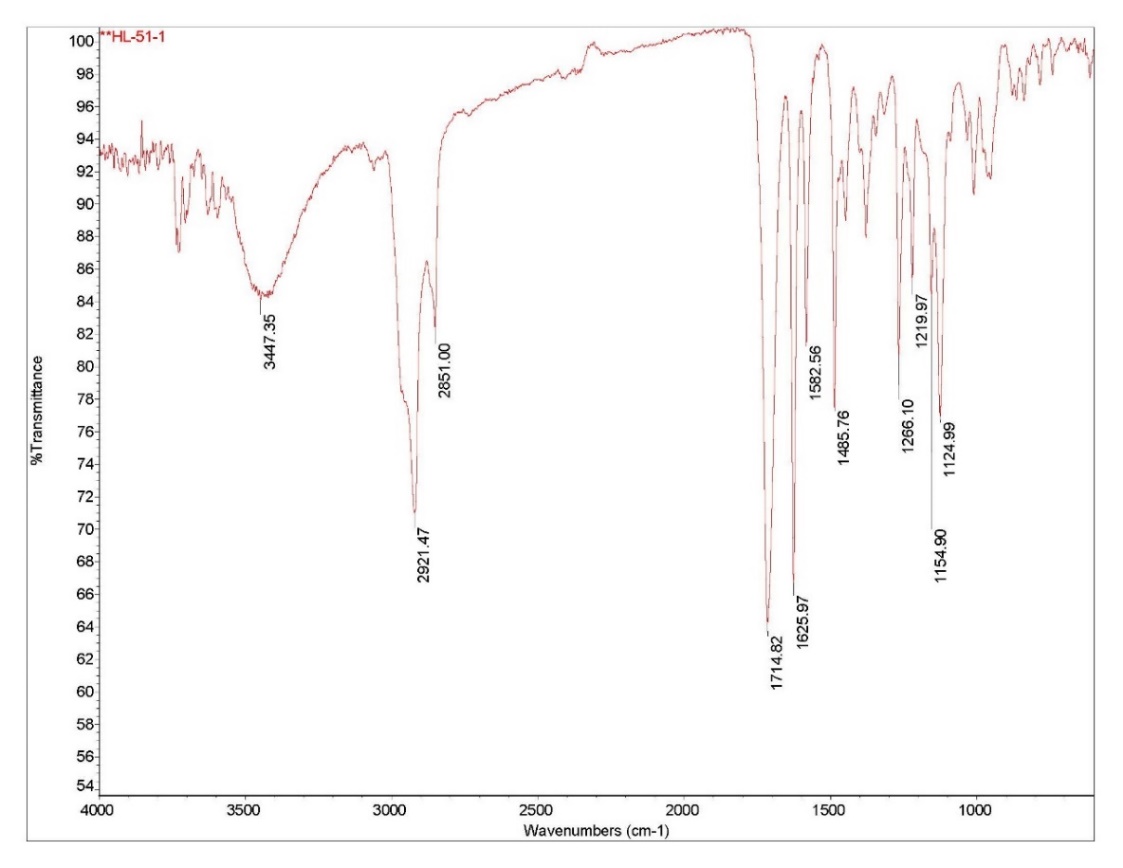


**Figure S15**. The IR spectrum of compound **2**.


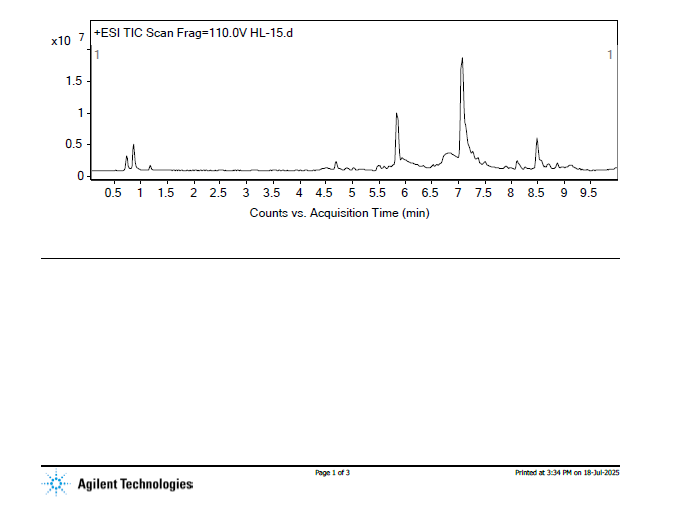


**Figure S16**. The (+)-HRESIMS report of compound **2**, page 1.


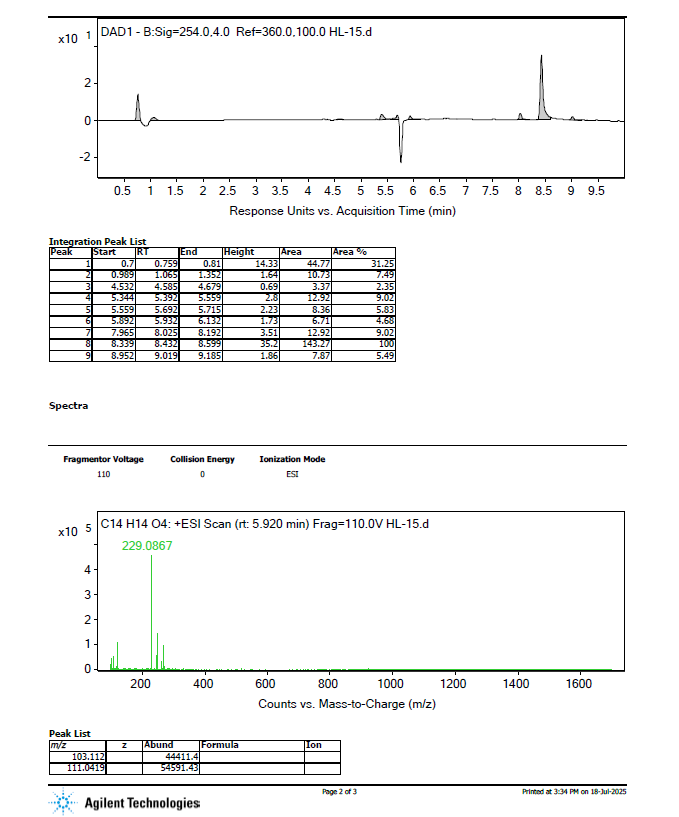


**Figure S17**. The (+)-HRESIMS report of compound **2**, page 2.


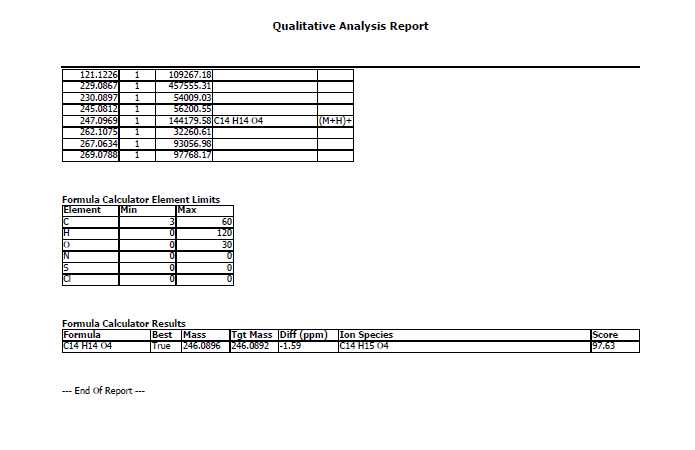


**Figure S18**. The (+)-HRESIMS report of compound **2**, page 3.


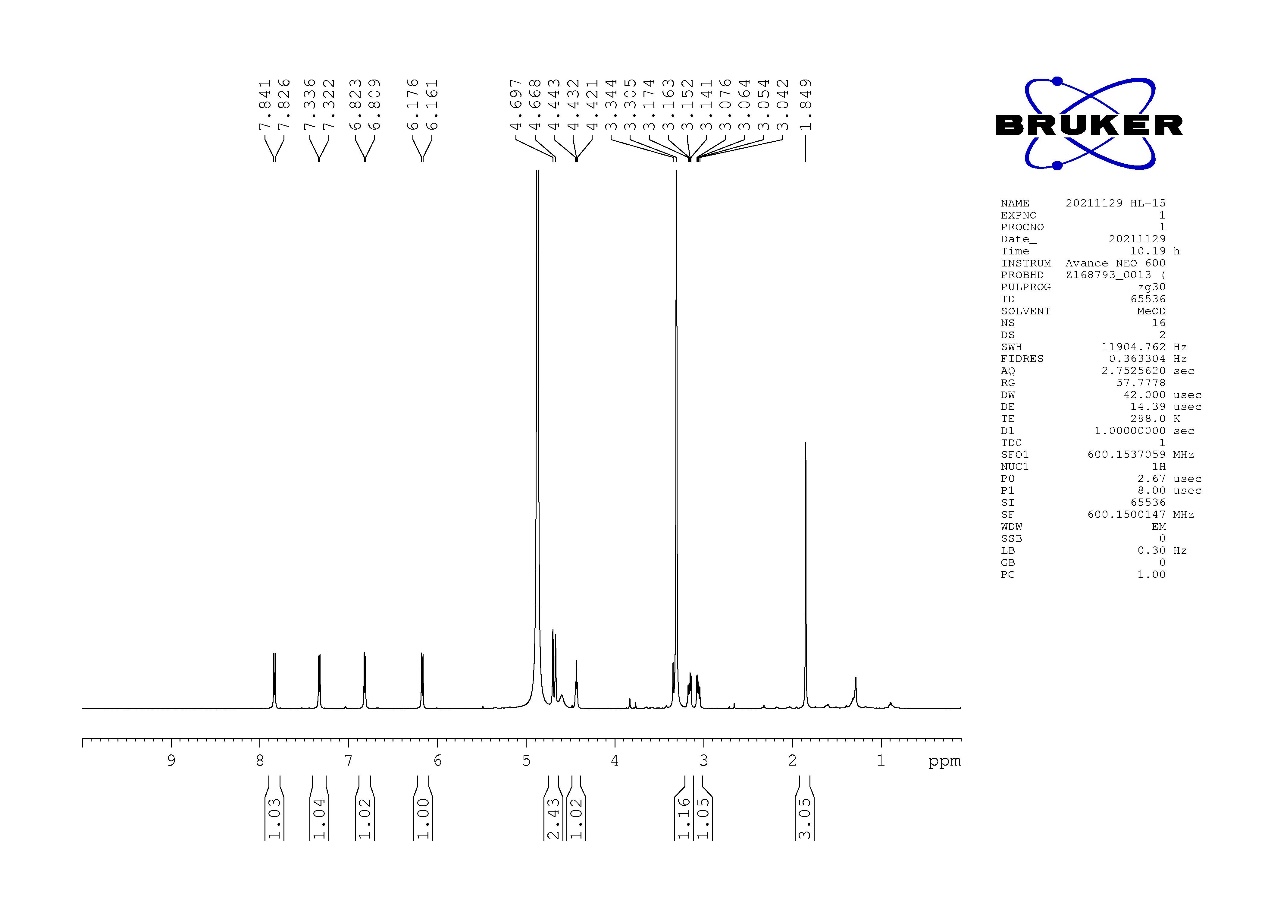


**Figure S19**. The ^1^H NMR spectrum of compound **2** in MeOH-*d*_4_ (600 MHz).


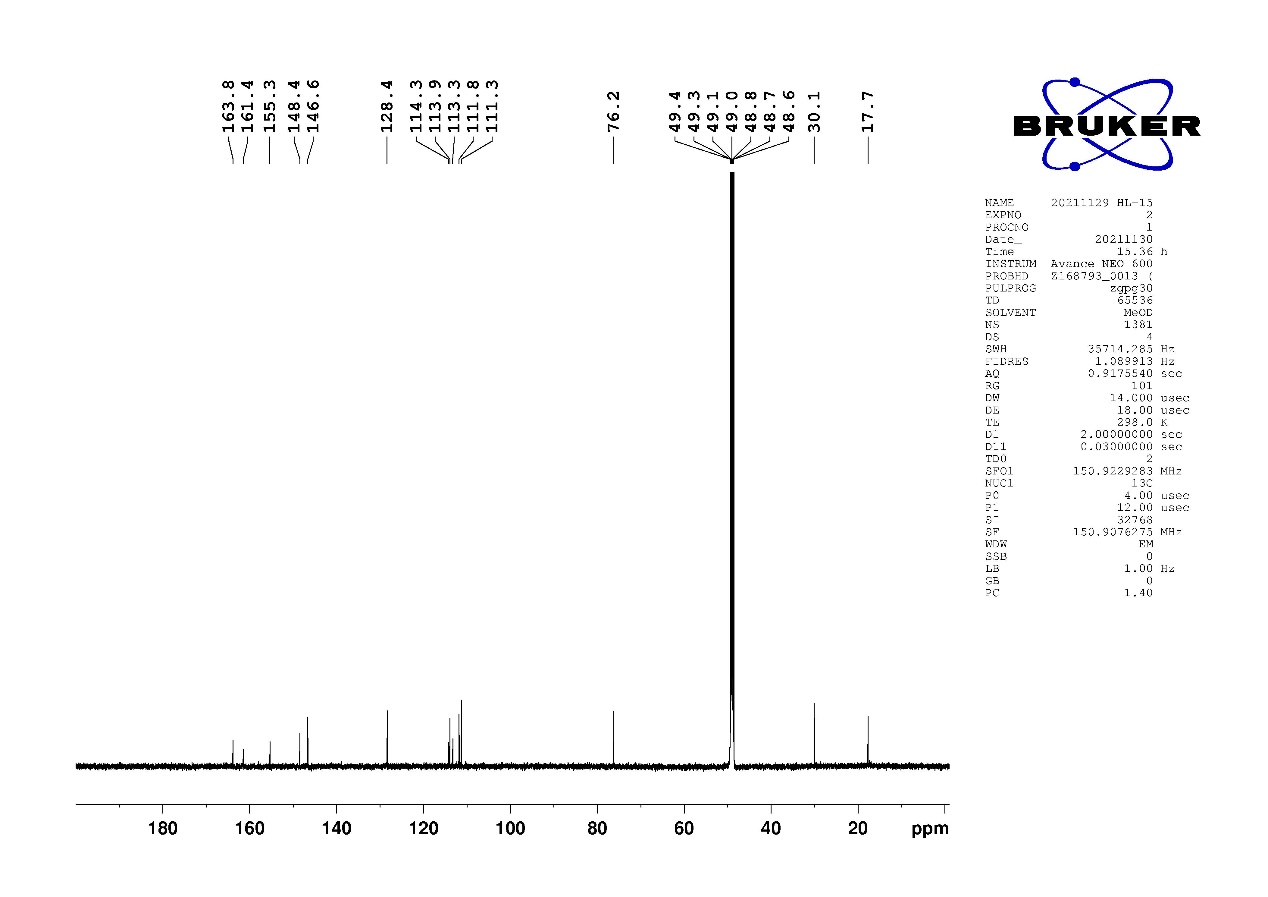


**Figure S20**. The ^13^C NMR spectrum of compound **2** in MeOH-*d*_4_ (150 MHz).


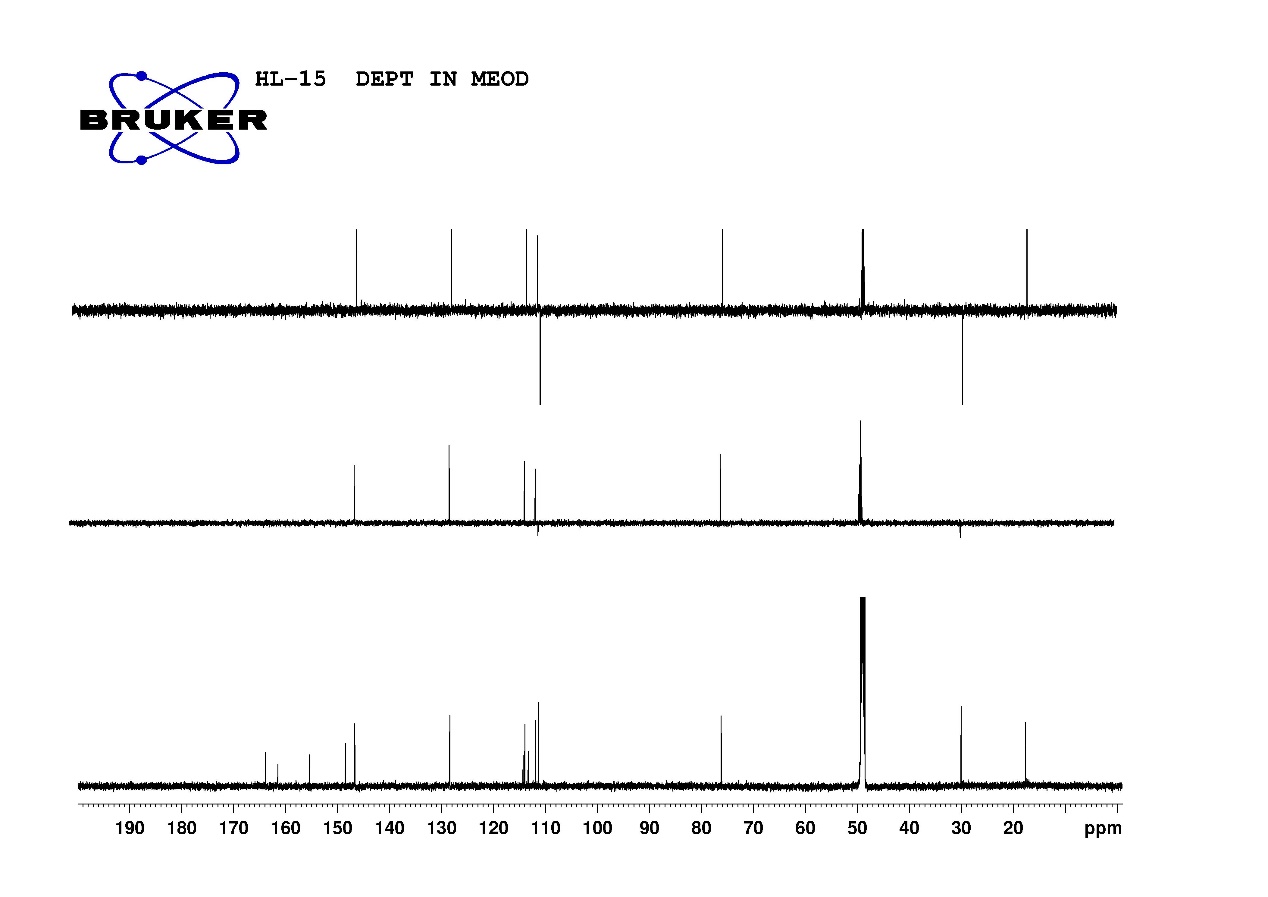


**Figure S21**. The DEPT spectrum of compound **2** in MeOH-*d*_4_ (150 MHz).


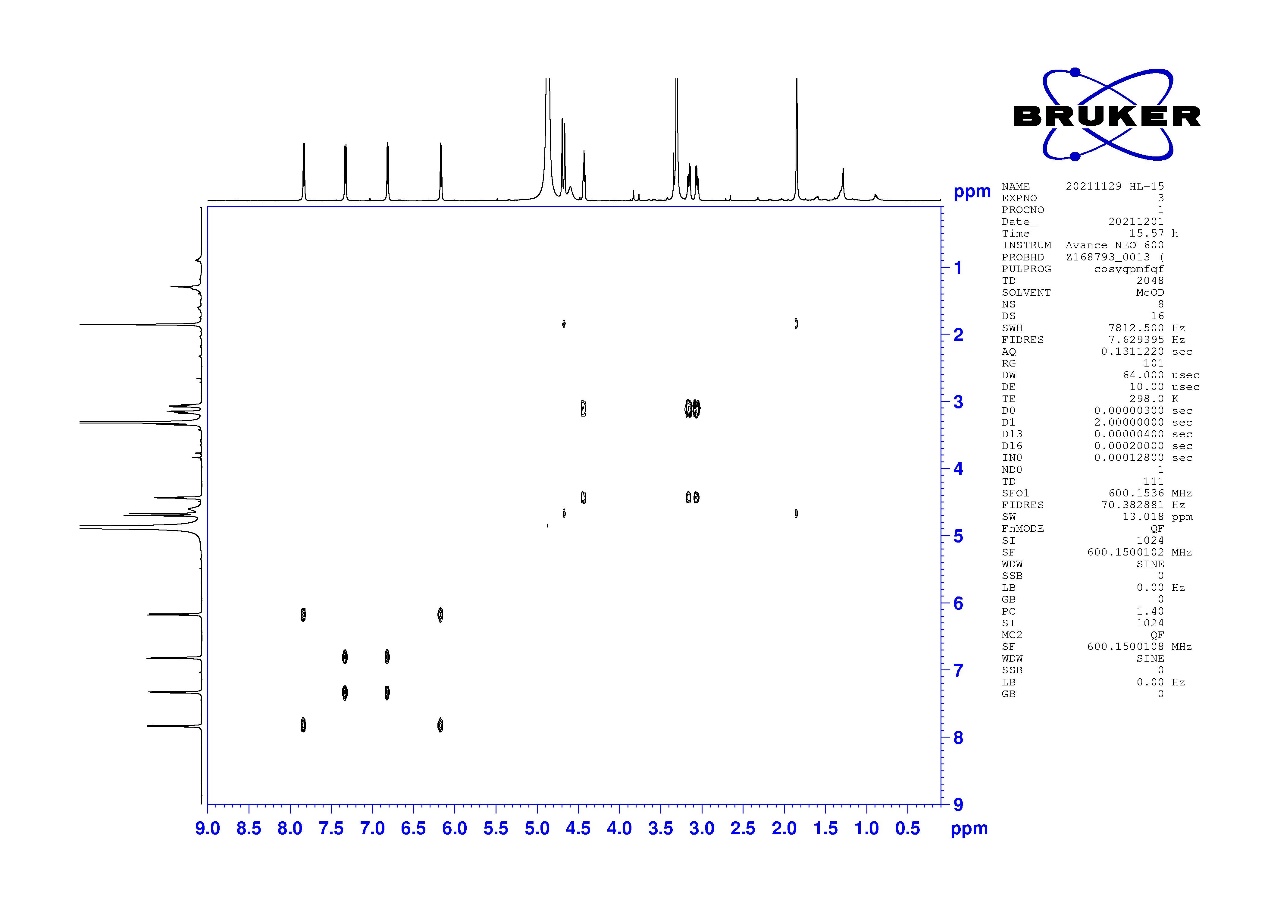


**Figure S22**. The ^1^H-^1^H COSY spectrum of compound **2** in MeOH-*d*_4_ (600 MHz).


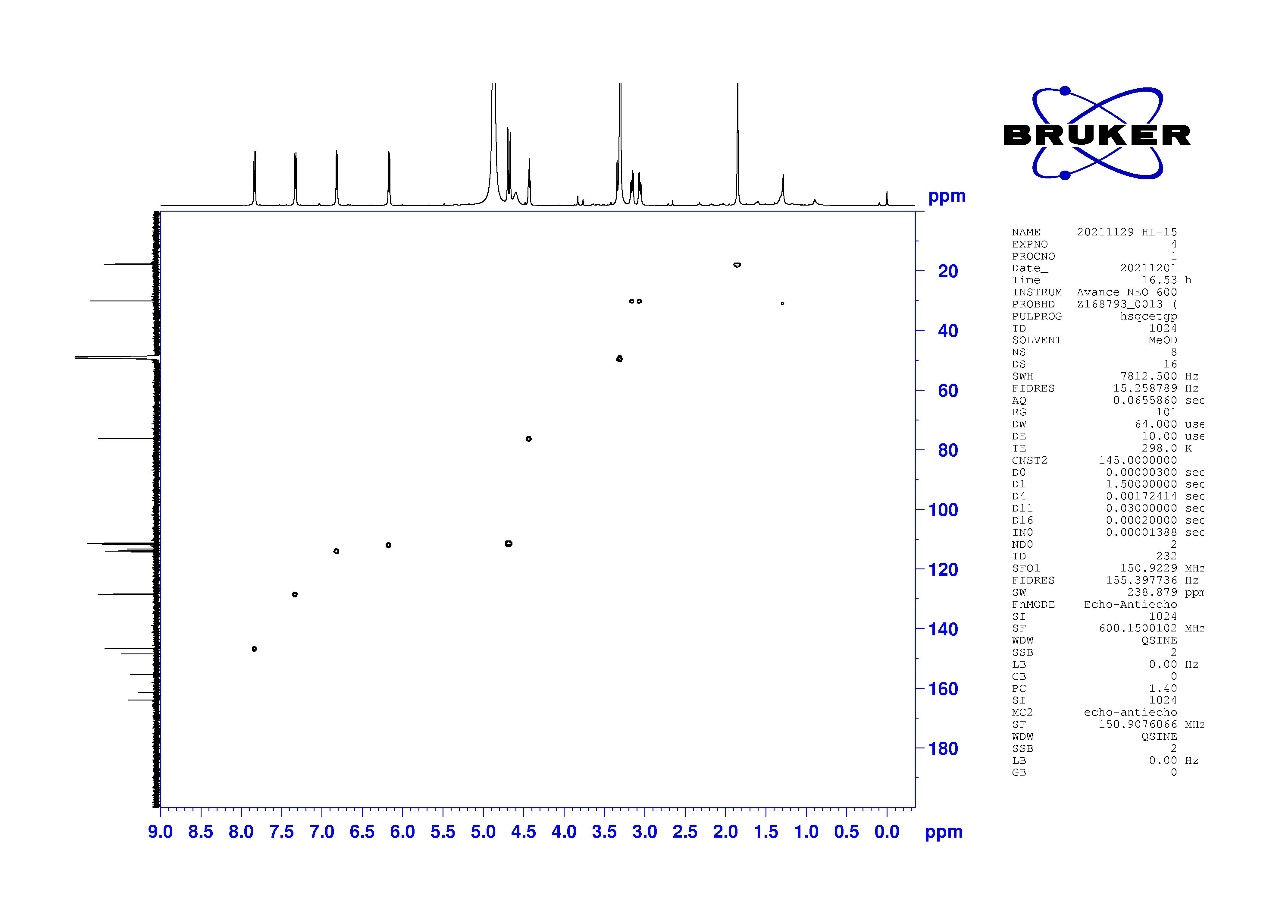


**Figure S23**. The HSQC spectrum of compound **2** in MeOH-*d*_4_ (600 MHz for ^1^H).


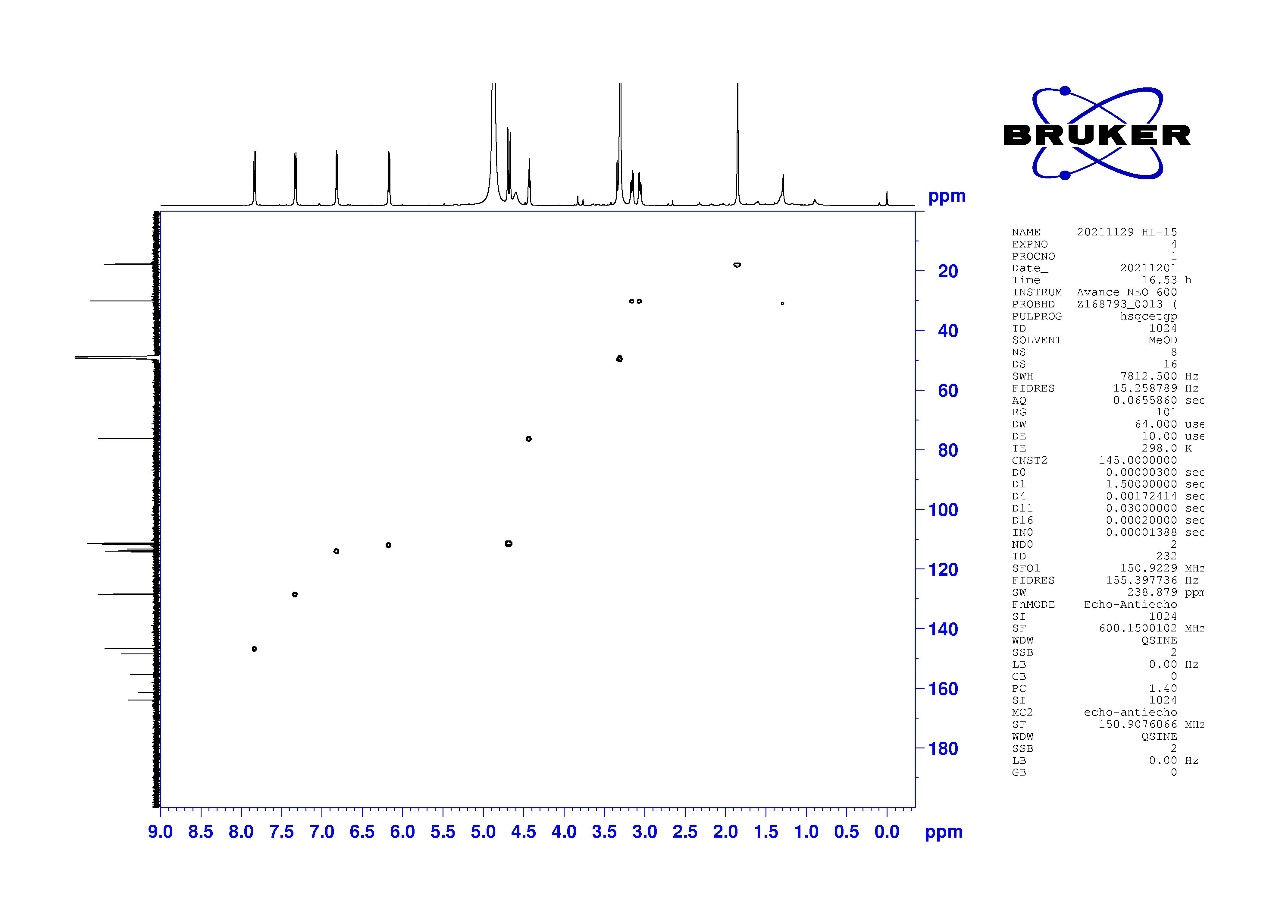


**Figure S24**. The HMBC spectrum of compound **2** in MeOH-*d*_4_ (600 MHz for ^1^H).

**Figure S25**. The chiral HPLC analysis of **3**.

**Figure S26**. The HPLC-UV spectrum of compound **3** in CH_3_CN/H_2_O.


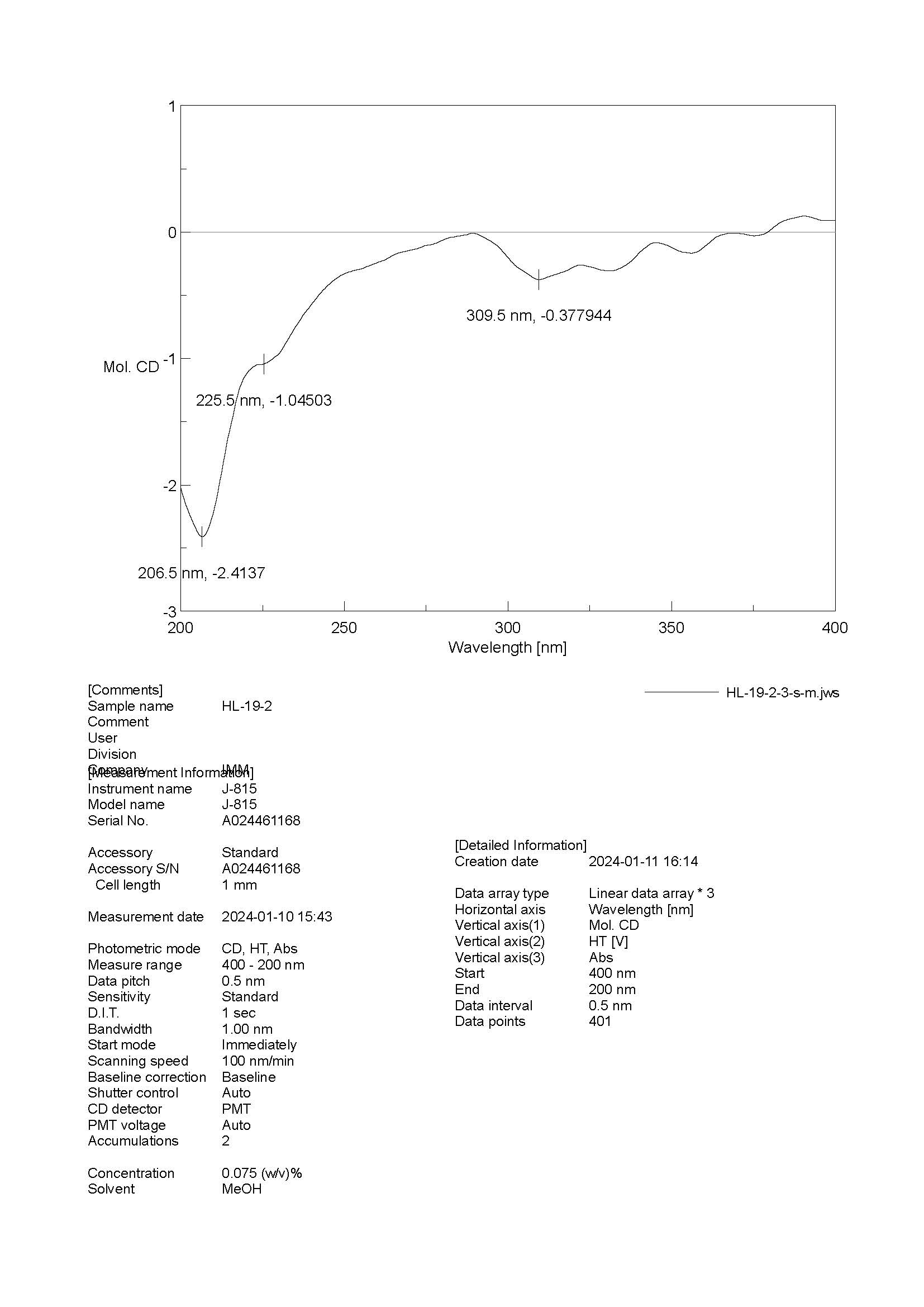


**Figure S27**. The CD spectrum of compound (+)-**3** in CH_3_OH.


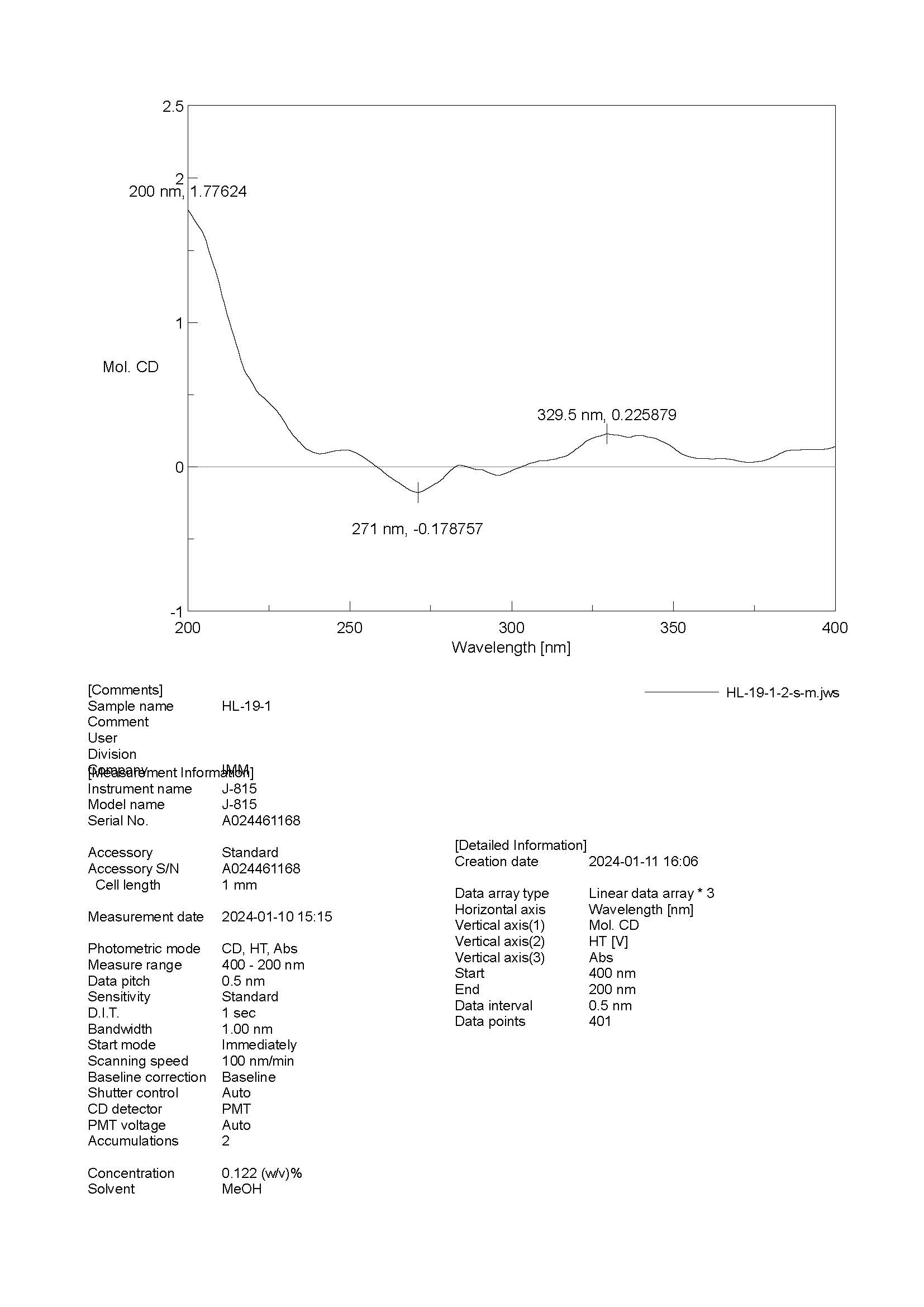


**Figure S28**. The CD spectrum of compound (–)-**3** in CH_3_OH.


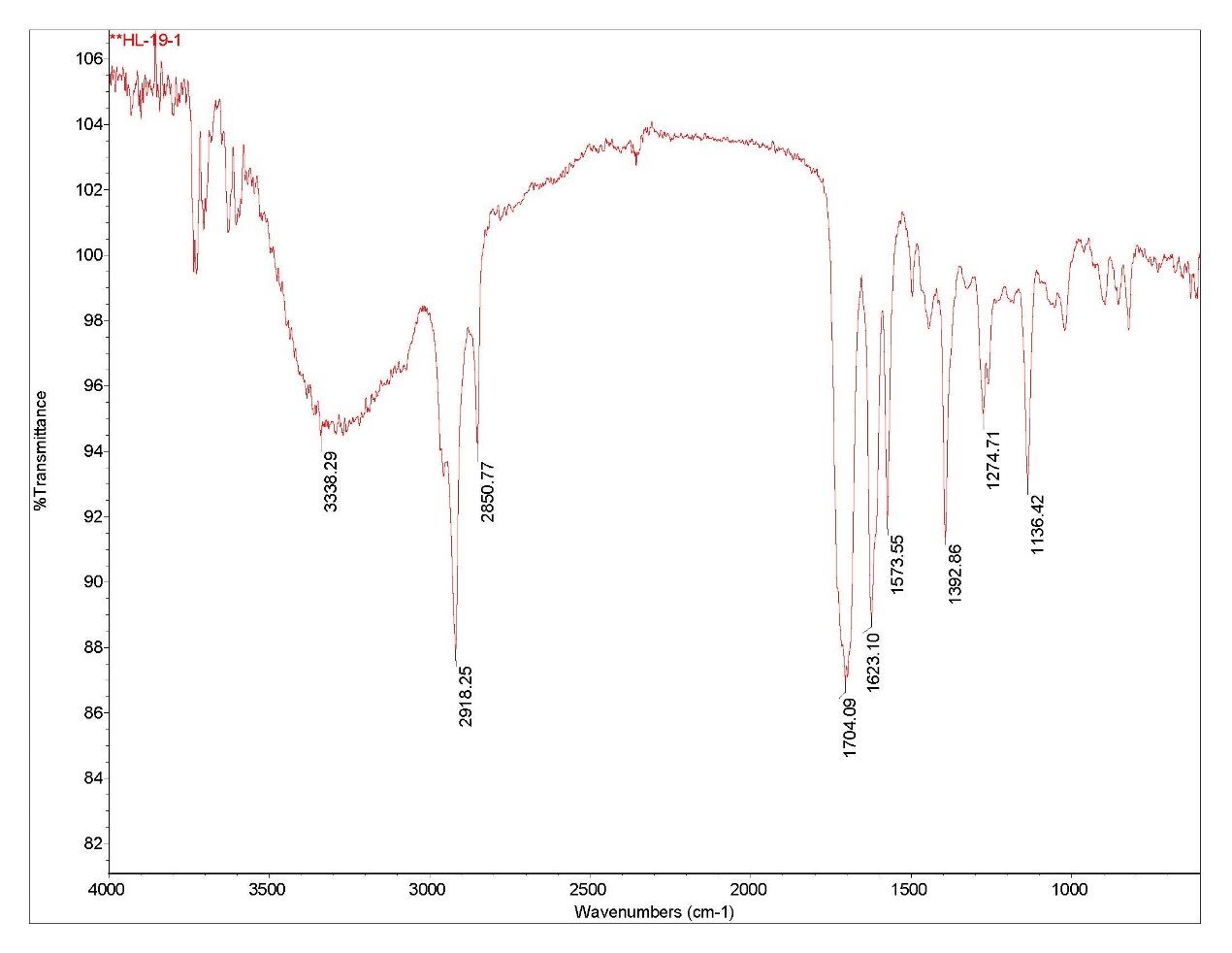


**Figure S29**. The IR spectrum of compound **3**.


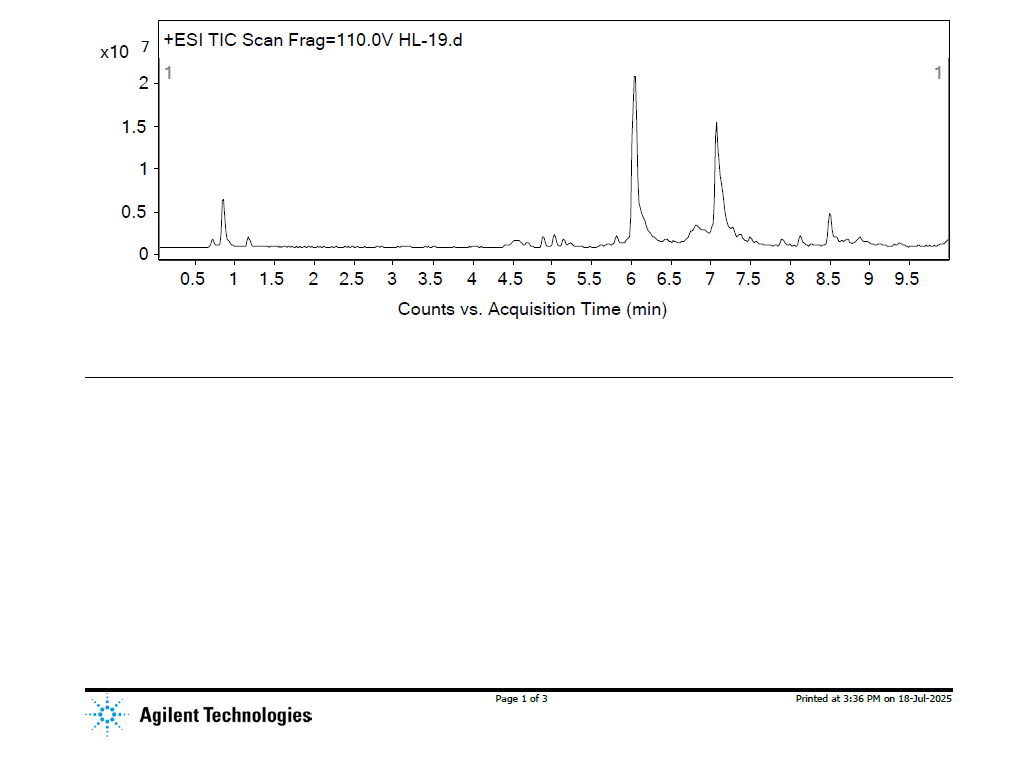


**Figure S30**. The (+)-HRESIMS report of compound **3**, page 1.


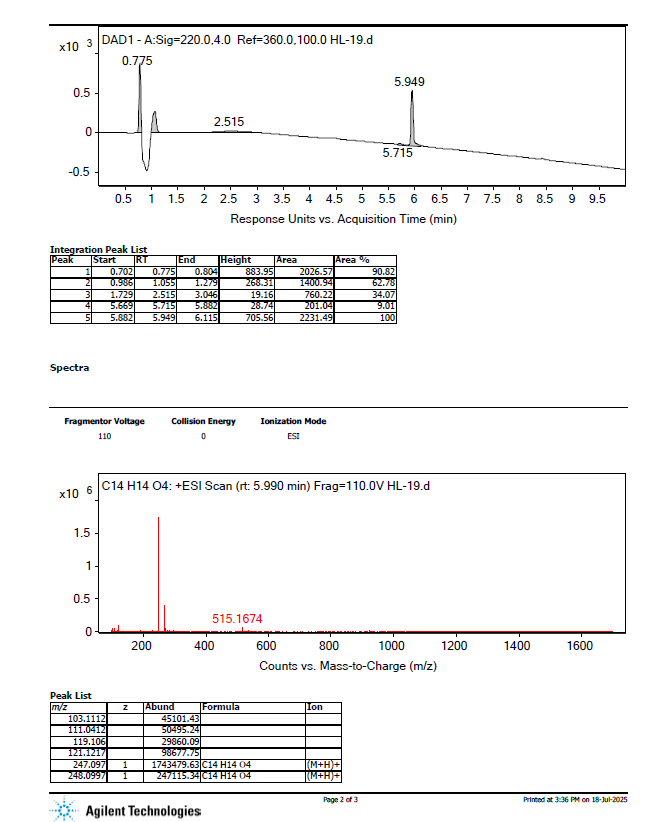


**Figure S31**. The (+)-HRESIMS report of compound **3**, page 2.


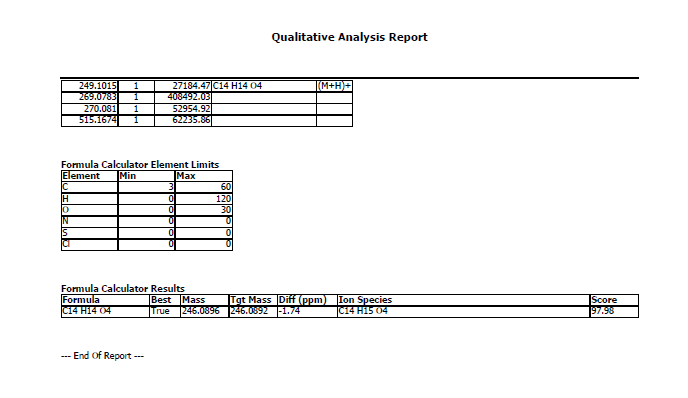


**Figure S32**. The (+)-HRESIMS report of compound **3**, page 3.


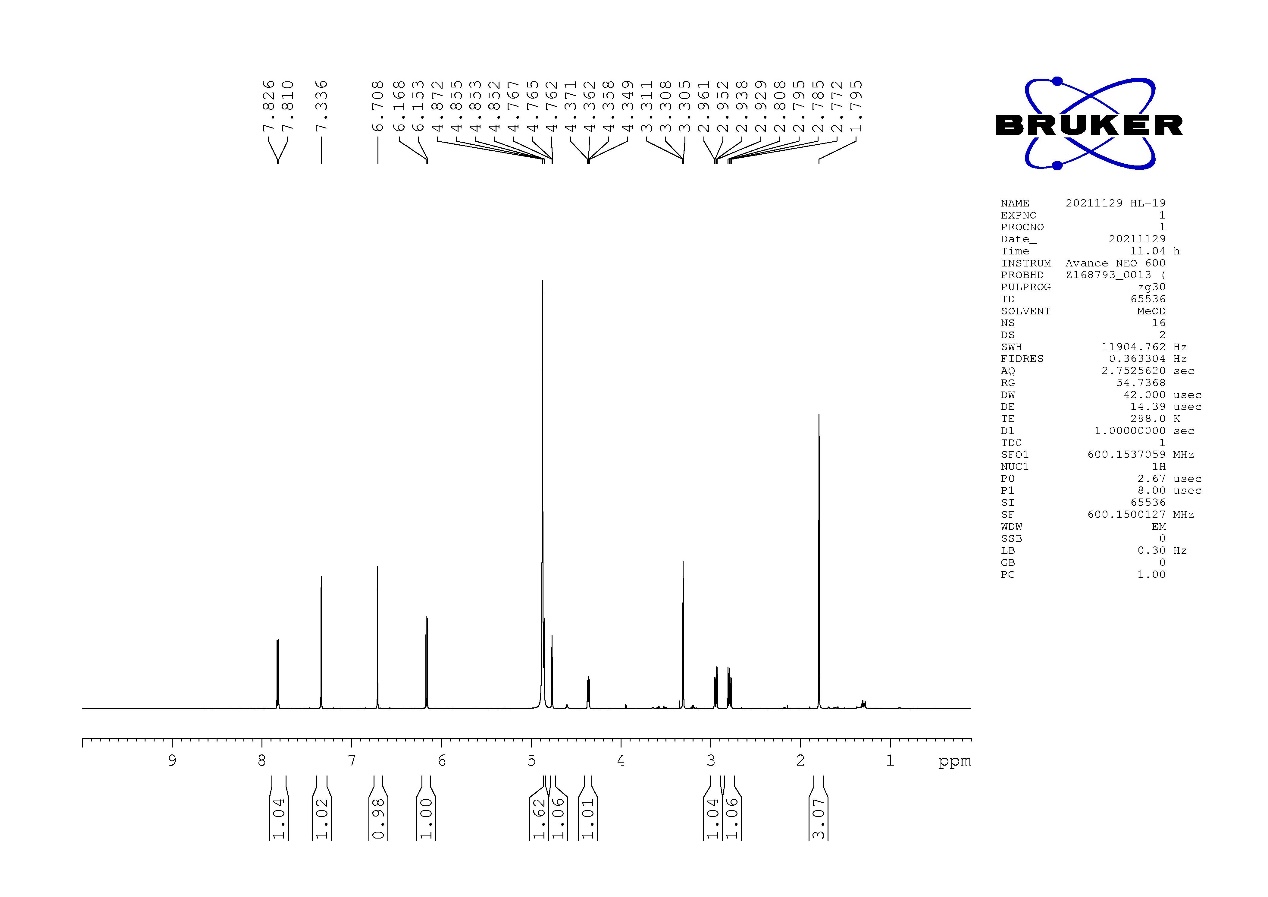


**Figure S33**. The ^1^H NMR spectrum of compound **3** in MeOH-*d*_4_ (600 MHz).


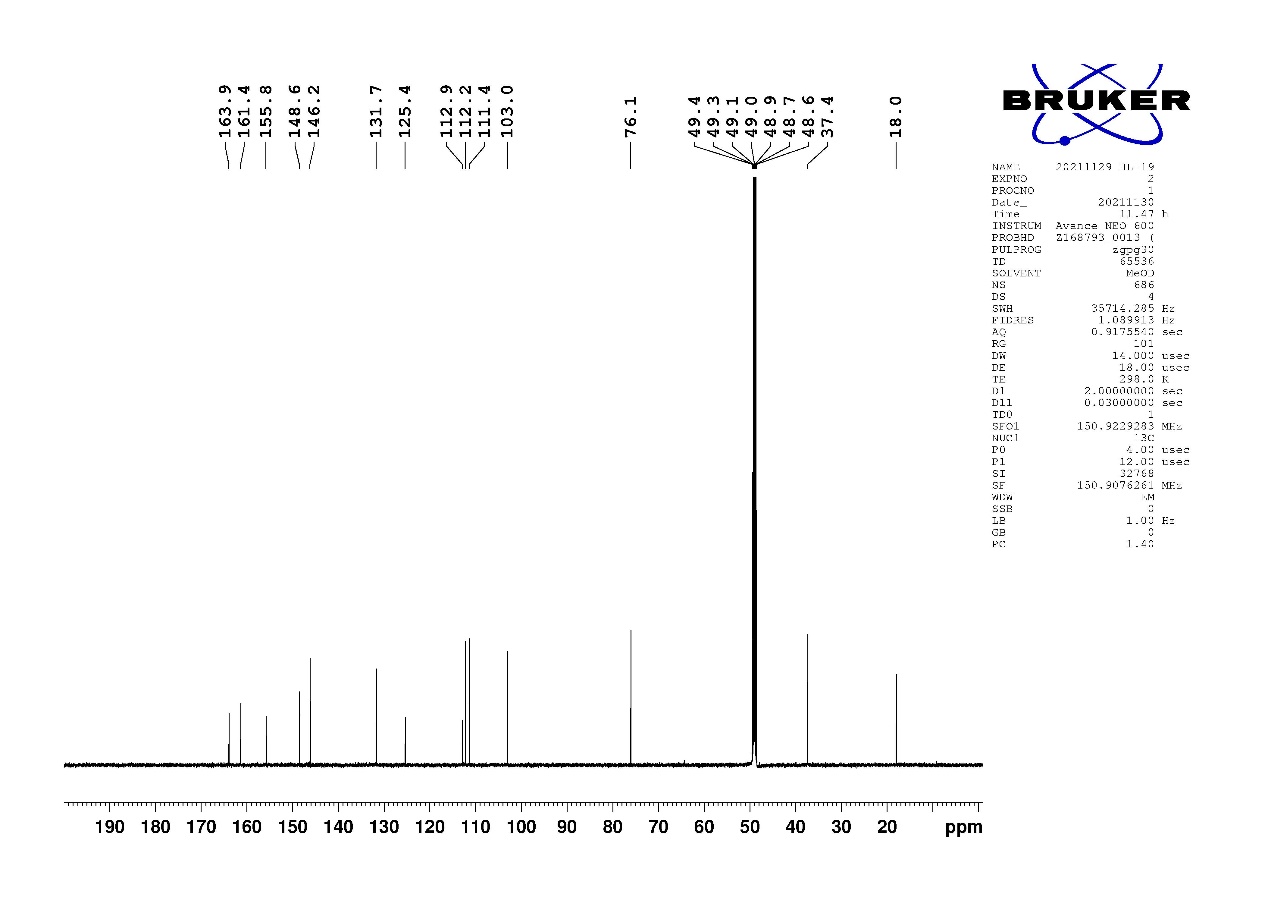


**Figure S34**. The ^13^C NMR spectrum of compound **3** in MeOH-*d*_4_ (150 MHz).
